# The sol–gel autocombustion as a route towards highly CO_2_-selective, active and long-term stable Cu/ZrO_2_ methanol steam reforming catalysts†

**DOI:** 10.1039/d1qm00641j

**Published:** 2021-05-20

**Authors:** Kevin Ploner, Parastoo Delir Kheyrollahi Nezhad, Albert Gili, Franz Kamutzki, Aleksander Gurlo, Andrew Doran, Pengfei Cao, Marc Heggen, Nicolas Köwitsch, Marc Armbrüster, Maximilian Watschinger, Bernhard Klötzer, Simon Penner

**Affiliations:** Department of Physical Chemistry, University of Innsbruck Innrain 52c A-6020 Innsbruck Austria simon.penner@uibk.ac.at +43 51250758003; Chair of Advanced Ceramic Materials, Institut für Werkstoffwissenschaften und -technologien, Technical University Berlin Hardenbergstr. 40 D-10623 Berlin Germany; Institute of Chemistry, Technical University Berlin Sekretariat TC 8 Straße des 17. Juni 124 D-10623 Berlin Germany; Advanced Light Source, Lawrence Berkeley National Laboratory Berkeley California 94720 USA; Ernst Ruska-Centre for Microscopy and Spectroscopy with Electrons, Forschungszentrum Jülich GmbH Leo-Brandt-Str. 1 D-52428 Jülich Germany; Faculty of Natural Sciences, Institute of Chemistry, Materials for Innovative Energy Concepts, Technical University Chemnitz Straße der Nationen 62 D-09111 Chemnitz Germany

## Abstract

The adaption of the sol–gel autocombustion method to the Cu/ZrO_2_ system opens new pathways for the specific optimisation of the activity, long-term stability and CO_2_ selectivity of methanol steam reforming (MSR) catalysts. Calcination of the same post-combustion precursor at 400 °C, 600 °C or 800 °C allows accessing Cu/ZrO_2_ interfaces of metallic Cu with either amorphous, tetragonal or monoclinic ZrO_2_, influencing the CO_2_ selectivity and the MSR activity distinctly different. While the CO_2_ selectivity is less affected, the impact of the post-combustion calcination temperature on the Cu and ZrO_2_ catalyst morphology is more pronounced. A porous and largely amorphous ZrO_2_ structure in the sample, characteristic for sol–gel autocombustion processes, is obtained at 400 °C. This directly translates into superior activity and long-term stability in MSR compared to Cu/tetragonal ZrO_2_ and Cu/monoclinic ZrO_2_ obtained by calcination at 600 °C and 800 °C. The morphology of the latter Cu/ZrO_2_ catalysts consists of much larger, agglomerated and non-porous crystalline particles. Based on aberration-corrected electron microscopy, we attribute the beneficial catalytic properties of the Cu/amorphous ZrO_2_ material partially to the enhanced sintering resistance of copper particles provided by the porous support morphology.

## Abstract 2

The adaption of the sol–gel autocombustion method to the Cu/ZrO_2_ system opens new pathways for the specific optimisation of the activity, long-term stability and CO_2_ selectivity of methanol steam reforming (MSR) catalysts.

## Graphical abstract

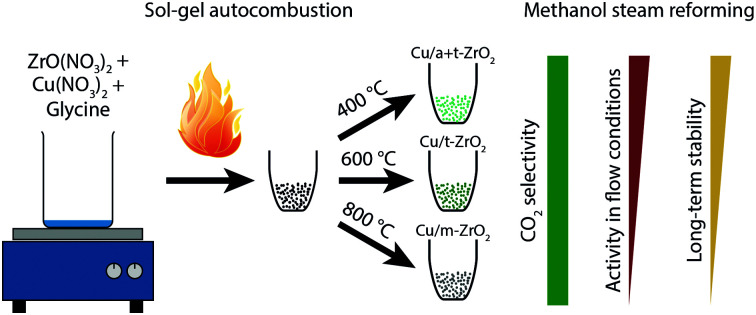

## 1. Introduction

Concerning renewable energy storage, methanol is a promising candidate due to its beneficial properties with respect to stocking and distribution.^1^ It is liquid at ambient conditions, has a high hydrogen-to-carbon ratio and the absence of a C–C bond considerably decreases the necessary temperature for selective reforming (*e.g.* CO_2_-selective steam reforming of methanol at 200–300 °C as opposed to ethanol steam reforming with inherently reduced CO_2_ selectivity at temperatures above 400 °C).^1–3^ Due to its high volumetric energy density, it is especially suitable for automotive applications, where the H_2_/CO_2_ reformate can be used in a polymer electrolyte membrane fuel cell (PEMFC).^1,4^ For this application, the concentration of CO has to be kept in the low ppm regime, as even small traces of CO can deteriorate the performance of PEMFC electrodes.^5^

The methanol conversion reaction exhibiting the highest hydrogen yield is stoichiometric methanol steam reforming^6^ (MSR):

1 CH_3_OH_(g)_ + H_2_O_(g)_ ⇄ 3H_2,(g)_ + CO_2,(g)_ Δ*H*^0^_r_ = 49.6 kJ mol^−1^

Several competing side reactions leading to the formation of either CO or CH_4_ need to be avoided. CO is either produced by methanol decomposition^6^ (eqn (2)) or the reverse water–gas shift reaction^6^ (eqn (3)):

2 CH_3_OH_(g)_ ⇄ 2H_2,(g)_ + CO_(g)_ Δ*H*^0^_r_ = 90.6 kJ mol^−1^

3 CO_2,(g)_ + H_2,(g)_ ⇄ H_2_O_(g)_ + CO_(g)_ Δ*H*^0^_r_ = 41.1 kJ mol^−1^

The occurrence of these side reactions induces further cleaning steps before employing the reformate in a PEMFC.^1,5,7^ CH_4_ can be formed by CO (eqn (4)) or CO_2_ (eqn (5)) methanation.^8^

4 CO_(g)_ + 3H_2,(g)_ ⇄ CH_4,(g)_ + H_2_O_(g)_ Δ*H*^0^_r_ = −206 kJ mol^−1^

5 CO_2,(g)_ + 4H_2,(g)_ ⇄ CH_4,(g)_ + 2H_2_O_(g)_ Δ*H*^0^_r_ = −165 kJ mol^−1^

PEMFC operation is typically not fully impaired by trace amounts of CH_4_, but the fuel efficiency is significantly decreased.

The archetypical MSR catalyst is commercially available Cu/ZnO/Al_2_O_3_.^2,7,9,10^ It suffers from various drawbacks, including significant deactivation by copper particle sintering, and features too high CO levels for direct use in a PEMFC.^5,7^ Therefore, alternative systems have to be developed fulfilling all requirements of an efficient MSR catalyst to render its use economically feasible.^1^

ZrO_2_ is a promising candidate as a synergistically active support for highly CO_2_-selective copper-based MSR catalysts.^2,11,12^ ZrO_2_ exhibits three crystal structures: monoclinic under ambient conditions (m-ZrO_2_, room temperature to 1170 °C, group *P*2_1_/*c*)^13^ and the high-temperature polymorphs tetragonal (t-)ZrO_2_ (1170–2370 °C, space group *P*4_2_/*nmc*)^14^ and cubic (c-)ZrO_2_ (2370–2680 °C, space group *Fm*3̄*m*),^15^ respectively. The latter two can be preserved as metastable structures at room temperature *via* particle size control or doping.^16–18^ Both Cu/m-ZrO_2_, as well as Cu/t-ZrO_2_ systems, were tested with conflicting reports in MSR. Examples of both well and undesirably performing catalysts with respect to CO_2_ selectivity in MSR are reported for Cu/m-ZrO_2_^19,20^ as well as Cu/t-ZrO_2_^11,12,19^ materials.

A comprehensive study on Cu/ZrO_2_ catalysts in MSR following dedicated synthesis routines revealed that especially the synthesis approach for the ZrO_2_ phase (determining its surface-chemical properties as the key parameter for a high CO_2_ selectivity) is crucial for the performance of Cu/ZrO_2_ catalysts in MSR. The influence of the type of copper precursor is limited.^19^ The surface-chemical (defect) behaviour of a specific ZrO_2_ polymorph could be steered by the synthesis pathway and the preparation history. Its bulk crystallographic structure is of minor importance for the MSR performance.^19^

Various synthesis approaches for MSR catalysts are reported in literature, aiming at the optimisation of the activity, CO_2_ selectivity and long-term stability.^2^ Among the most prominent ones are wet impregnation (Cu/ZrO_2_,^19^ Cu/Zn + Cu/Cr + CuZr on Al_2_O_3_,^21^ Cu/Al_2_O_3_ + Zn and Ce,^22^ CeO_2_- + ZrO_2_-promoted Cu/ZnO on Al_2_O_3_^23^) and co-precipitation (Cu/ZrO_2_,^19^ CuO/CeO_2_,^24^ ZrO_2_- and Al_2_O_3_-promoted Cu/ZnO,^25^ Cu/CeO_2_^26^), but also other less commonly utilised methods like hydrothermal synthesis (Cu/Zn/Al^27^), soft reactive grinding (Cu_1.5_Mn_1.5_O_4_ spinel^28^), oxalate gel co-precipitation (Cu/MnO_*x*_,^28^ Cu/ZrO_2_,^29^ Cu/ZrO_2_^30^), the polymer template sol–gel method (Cu/ZrO_2_^12^) and urea nitrate combustion (Cu/CeO_2_,^31^ Cu/CeO_2_ doped with Sm, Zn, La, Zr, Mg, Gd, Y, Ca^32^) were employed. Each of these syntheses offers certain advantages, but alternative methods satisfying all prerequisites of an ideal MSR catalyst are still to be developed.

One promising candidate is the sol–gel autocombustion method – the urea nitrate combustion is a subgroup of this method – that grants access to homogeneous oxide powders by the use of complexing agents like glycine or urea to prevent selective precipitation of metal ions during the removal of water.^33^ At the same time, they act as the fuel for the autocombustion of the correspondingly employed metal nitrates, yielding finely dispersed oxidised powders. Additionally, the sol–gel autocombustion method offers the advantages of simplicity as a one-step approach, a high versatility concerning the educts and a reasonably high surface area.^33^

The sol–gel autocombustion technique can be utilised for the synthesis of many material classes and is primarily employed for perovskite or spinel phases with various applications (CoFe_2_O_4_^34^ and Li_0.5_Fe_2.5_O_4_^35^ with special magnetic properties, (CeO_2_)_0.9_(SmO_1.5_)_0.1_^36^ as electrolyte in a solid oxide fuel cell, LaMnO_3_^37^ for the oxygen reduction reaction, LaFeO_3_^38^ as well as doped La(Cu_0.7_Mn_0.3_)O_3_^39^ for the reduction of NO by CO), but also for other catalysts, *e.g.* NiO/ZrO_2_^40^ for chemical-looping combustion. For the preparation of catalysts for MSR, this method was mainly used for Cu/ZnO/Al_2_O_3_ systems and different variants obtained by substitution/addition of selected components.^41–45^ All of these systems display a specific morphology featuring so-called combustion pores that are formed by the expansion of the gases produced during the reaction of the fuel and the oxidizing agent.^43,44^ One particular CuO/ZnO/Al_2_O_3_ system prepared with ethylene glycol as the fuel displayed a high activity and long-term stability in MSR, which is explained by a high number of combustion pores and a small particle size.^44^ Yu *et al.*^46^ were successful in the preparation of a highly active and stable CuFeO_2_–CeO_2_ MSR catalyst *via* an analogous sol–gel autocombustion routine.

For binary Cu/ZrO_2_ MSR catalysts, specific wet impregnation,^19,29^ co-precipitation,^19,29^ oxalate gel co-precipitation,^29,30^ the polymer template sol–gel method^12^ and conventional sol–gel methods^47^ have been employed. Wet impregnation is a comparably easy synthesis route, but the resulting Cu/ZrO_2_ catalysts can suffer from elevated deactivation in MSR.^19^ Similar observations concerning long-term stability have been made for aqueous co-precipitation,^29^ while more sophisticated techniques like oxalate gel co-precipitation^29^ and the polymer template sol–gel method^12^ alleviate these stability issues. Conventional sol–gel methods provide exceptionally high surface areas and copper dispersion,^47^ but the latter three approaches all consist of multiple synthesis steps, making the process more complex and costly.

In the present work, the sol–gel autocombustion method is adapted to obtain binary Cu/ZrO_2_ catalysts for methanol steam reforming, making use of a number of advantages, which potentially lead to highly active and CO_2_-selective catalyst materials. Apart from the rather simple one-step process and the generally high versatility of educts, the high dispersion of metal ions and the characteristic morphology featuring combustion pores lead to a high number of catalytically active sites. Characterisation of the catalysts by *in situ* X-ray diffraction studies during the post-combustion calcination in combination with aberration-corrected electron microscopy allows us to directly follow the development of the bulk and particle structures of the active Cu/ZrO_2_ phase and to directly relate the obtained morphology to activity and CO_2_ selectivity under MSR operation. For the latter, we correlate batch reactor studies, detailing the development of different trace products as a function of pre-MSR calcination temperature, with long-term stability tests in a flow reactor.

## 2. Experimental

### 2.1. Sample synthesis

The catalysts consisting of ≈10 mol% Cu (exact loading is difficult to reach because of unknown water content of ZrO(NO_3_)_2_·*x*H_2_O; 10 mol% Cu correspond to 5.4 wt% Cu to ensure comparability with other systems from our workgroup^19^) supported on ZrO_2_ were prepared using the sol–gel autocombustion method.^33^ Cu(NO_3_)_2_·3H_2_O (≈0.03 mol l^−1^ in water, Merck, Darmstadt, ≥99.5%) and ZrO(NO_3_)_2_·*x*H_2_O (≈0.25 mol l^−1^ in water, Alfa Aesar, Puratronic, 99.994% metals basis) were separately dissolved in deionised water and subsequently added to an aqueous solution of glycine (≈0.56 mol l^−1^ in water, Sigma Aldrich, ≥99%). This yields a NH_2_:NO_3_^−^ ratio being slightly larger than equimolar and final concentrations of 0.01 mol l^−1^ Cu(NO_3_)_2_·3H_2_O, 0.08 mol l^−1^ ZrO(NO_3_)_2_·*x*H_2_O and 0.19 mol l^−1^ glycine. The blueish solution was stirred at room temperature for 30 min and subsequently, the temperature was increased to 90–95 °C to evaporate the water. Over the course of 3 h, the solution transformed into a viscous blue gel. This gel was heated in a beaker to 250 °C in air, where the metal oxides, as well as carbon residues, were formed in a self-ignited combustion. Calcination at 400 °C, 600 °C and 800 °C for 2 h removed the remaining carbon, indicated by the change of the initially black colour, with simultaneous formation of either a mostly amorphous, a tetragonal or a monoclinic polymorph of ZrO_2_ (*cf.* ESI,† Fig. S7). The nominal Cu loading has been verified by dedicated TGA oxidation–reduction experiments. The latter have been proven to yield very accurate values of the Cu content, especially for Cu/ZrO_2_ catalysts.^19^

### 2.2. Catalytic characterisation

#### 2.2.1. Recirculating batch reactor

The heated sample-containing part of the recirculating all-quartz glass batch reactor can be heated with a Linn High Term furnace up to 1100 °C. The temperature is monitored by a K-type thermocouple (NiCr–Ni) close to the sample. The reactor compartment is capillary-connected to a quadrupole mass spectrometer (QMS, Balzers QMG 311) for continuous quantification of the gas phase composition, creating a quasi-closed system due to the small gas withdrawal rate. The QMS is equipped with a secondary electron multiplier and a cross-beam ion source. The recirculating batch reactor is specialised for the characterisation of small sample amounts (10–100 mg) and the quantification of trace by-products owing to its small reactor volume (13.8 ml).

The MSR mixture is provided as a liquid solution of methanol and water in a ratio to yield an equilibrium gas phase composition of methanol : water = 1 : 2 at room temperature. The mixture and the gas phase are initially purified with three freeze–pump–thaw cycles before the gas phase is expanded into the pre-evacuated reactor. As standard pre-treatments for MSR catalysts, oxidative cleaning in pure O_2_ at 400 °C for 1 h (termed O400) and pre-reduction in pure H_2_ at 300 °C for 1 h (termed H300) are conducted before the methanol steam reforming experiment. For MSR, the reaction mixture (≈28 mbar) is introduced into the reactor at 100 °C (to prevent condensation on the sample) and Ar is added to correct the mass traces of the reactants and products with respect to thermal expansion during heating and the slow gas withdrawal to the QMS vacuum chamber through the capillary. Helium is added as a carrier gas up to atmospheric pressure, which enhances the heat transfer to and in the catalyst bed, as well as the recirculation efficiency. After an equilibration period of 40 min, the temperature program is started and the gas-phase composition is quantified *via* the QMS. Baseline and Ar intensity correction in combination with external calibration of the relevant gases including their relative fragmentation patterns (*e.g.* for correction of the *m*/*z* = 28 fragment of CO_2_) yields the evolution of the effective temperature- and total pressure drop-corrected partial pressures of the gaseous species.

To obtain the formation rates in terms of specific activities, the partial pressures are differentiated with respect to the time and converted to molar amounts utilizing the ideal gas law. Normalisation to the copper mass yields the specific activity in μmol g_Cu_^−1^ s^−1^. The methanol conversion (*x*_MeOH_) is obtained as a relative value by relating the *m*/*z* = 31 signal at each time to the value at the start of the temperature ramp (eqn (6)). The accuracy of the methanol conversion was estimated utilizing the standard deviation of the constant *m*/*z* = 31 signal at its highest point in a reference measurement, as the noise scales with the total signal height. This standard deviation was multiplied by 3 and propagated to the accuracy of the methanol conversion according to eqn (6), yielding a maximum accuracy of ≈0.01 (≈1%) at low methanol conversions, which becomes smaller at higher conversions.6
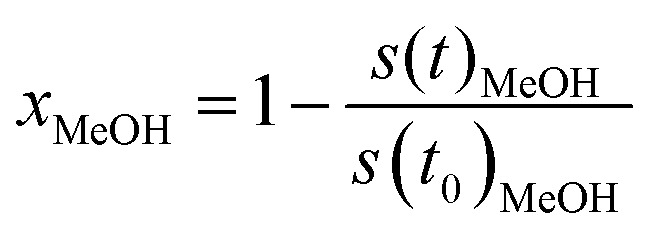
with *x*_MeOH_ = methanol conversion, *s*(*t*)_MeOH_ = *m*/*z* = 31 signal at time *t* (in a.u.), *s*(*t*_0_)_MeOH_ = *m*/*z* = 31 signal at the start of the temperature program (in a.u.).

The integral CO_2_ selectivity is obtained by division of the partial pressure of CO_2_ by the sum of the values of CO, CO_2_ and CH_4_. Values larger than 1 caused by slight deviations in the baseline (when the sum of the partial pressures is close to 0, division by it causes huge artefacts in the integral CO_2_ selectivity) were manually set to 1.

The apparent activation energy of CO_2_ formation *E*_A_(CO_2_) was calculated by fitting an Arrhenius function to the specific activity plotted *vs.* the absolute temperature at the beginning of the rate increase at a methanol conversion below 10%. Simultaneous variation of both *E*_A_ and the pre-exponential factor *A* yields values ranging from 1 × 10^7^ to 5 × 10^9^ μmol g_Cu_^−1^ s^−1^ for the latter. Hence, a fixed average value of 1 × 10^8^ μmol g_Cu_^−1^ s^−1^ is employed in the fits to enhance the relative comparability of the related activation energies.

#### 2.2.2. Continuous flow reactor

For the continuous flow MSR tests, a fixed bed reactor system (PID Eng&Tech, Microactivity Reference) connected to a MicroGC (Varian CP 4900, equipped with a 10 m back-flushed M5A column, a 20 m back-flushed M5A column and a 10 m PPU column) for the simultaneous analysis of H_2_, CH_4_, CO and CO_2_ was employed. To ensure a homogenous gas flow through the sample, the catalysts were diluted by mixing them with catalytically inert graphite (ChemPur, <100 μm, 99.9%). Then, they were placed in the reactor tube (stainless steel coated with silicon oxide, inner diameter 7.9 mm) on top of a quartz-glass fleece. For the MSR tests, a mixture of 10% He/N_2_ (15 ml min^−1^, GHSV = 1925 h^−1^, Air Liquide, 99.999%) was used as carrier gas, which was loaded with a stoichiometric 1 : 1 H_2_O/MeOH vapour mixture (*via* simultaneous evaporation of 0.01 ml min^−1^ H_2_O_(l)_, 0.0225 ml min^−1^ MeOH_(l)_, Fisher Scientific, HPLC grade). The unconverted fraction of the reactant vapours was condensed downstream in a cooling trap and the gas stream was further dried by a Nafion® membrane, which was dried in counter flow by a N_2_-flow of 100 ml min^−1^. Finally, the dry gas stream was analysed by online gas chromatography to determine the specific activity using the ideal gas law and normalisation to the copper mass. The CO_2_ selectivity is obtained by division of the specific activity of CO_2_ by the sum of the values of CO, CO_2_ and CH_4_.

### 2.3. *In situ* X-ray diffraction (*in situ* XRD)

The bulk structural changes of the post-combustion powders of ZrO_2_ and Cu/ZrO_2_ were investigated with temperature-resolved *in situ* synchrotron XRD measurements at the beamline 12.2.2 at the Advanced Light Source (ALS) at the Lawrence Berkeley National Laboratory (LBNL), California, in a setup previously described by Doran *et al.*^48^ and Schlicker *et al.*^49^ A monochromatic beam with an energy of 25 keV and a spot size of 30 μm was utilised in transmission mode and the diffraction pattern was recorded with a PerkinElmer XRD 1621 image plate detector, collecting one pattern per 60 s. A LaB_6_ NIST standard was measured for the calibration of the sample-to-detector distance, the detector tilt and the exact wavelength (0.4959 Å) using the Dioptas software,^50^ which was also utilised for the conversion of the 2D detector images to diffraction patterns by radial integration.

The sample powder (≈1 mg) was placed in a quartz capillary with a diameter of 700 μm located inside a SiC sleeve that was heated with two infrared lights.^48^ A flow of 2 ml min^−1^ O_2_ and 8 ml min^−1^ Ar was supplied by Alicat mass flow controllers and a sequence of heating with a rate of 5 °C min^−1^ from 25–800 °C, an isothermal period of 20 min and cooling to room temperature with 20 °C min^−1^ was executed.^49^

### 2.4. Scanning transmission electron microscopy (STEM)

Scanning transmission electron microscopy (STEM) was performed using a FEI Titan 80–200 (ChemiSTEM) electron microscope with a C_s_-probe corrector (CEOS GmbH) and a high-angle annular dark field (HAADF) detector. The microscope was operated at 200 kV. To achieve “Z-Contrast” conditions, a probe semi-angle of 24.7 mrad and an inner collection semi-angle of the detector of 88 mrad were used. Compositional maps were obtained with energy-dispersive X-ray (EDX) spectroscopy using four large-solid-angle symmetrical Si drift detectors. For EDX elemental mapping, Cu K and Zr K peaks were used.

### 2.5. N_2_ adsorption according to the BET method

The specific surface area was measured using a NOVA 2000e Surface Area & Pore Size Analyzer (Quantachrome Instruments) and the software Quantachrome NovaWin. Prior to the adsorption of N_2_ at −196 °C, the samples were heated *in vacuo* under active pumping at 200 °C for 1 h to remove any adsorbed water. The adsorption isotherm was measured at five points from 0.05 to 0.30 *p*/*p*_0_ and evaluated according to the BET model.^51^

### 2.6. Dissociative N_2_O adsorption

The determination of the accessible specific copper surface area is based on the selective oxidation of metallic Cu to Cu_2_O at the surface using N_2_O.^52–54^ The measurements were conducted in a quartz-glass reactor with a volume (including the sample) that is precisely calibrated by expansion of He (5.0, Messer) from a manifold with a defined volume. To account for the temperature gradient in the reactor, this calibration is performed at a sample temperature of 300 °C. To prevent the contribution of water formed by the reduction to the total pressure, a degassed zeolite trap is placed in the cold zone of the reactor. Since only metallic copper can be oxidised by N_2_O, the calcined samples are pre-reduced in pure flowing H_2_ (5.0, Messer) at 300 °C for 30 min. Then, the reactor is evacuated, purged with He and evacuated once more, before the sample is cooled down to 70 °C and subjected to a defined pressure of N_2_O (2.0, Messer) measured by a Baratron pressure transducer (MKS Instruments). A quadrupole mass spectrometer (QMS, Balzers QMA125 + QME 125-9) was employed for monitoring the formation of N_2_ and the consumption of N_2_O for 90 min. Following evacuation and flushing with He, the temperature was increased to 300 °C *in vacuo*. Thereafter, a defined pressure of H_2_ was provided in the manifold, which can be converted to a molar amount utilizing the ideal gas law. After opening the valve to the sample, the pressure decrease caused by the reduction of the N_2_O-induced surface Cu_2_O layer was monitored for 35 min. Based on the preceding He calibration and the thereby calculated effective volume at 300 °C, the molar amount *n*_H_2__ of consumed H_2_ can be calculated *via* the ideal gas law. Therefore, the remaining molar amount of H_2_ in the effective volume *V*_eff_ at 300 °C is subtracted from the supplied molar amount of H_2_ in the manifold at room temperature before expansion (eqn (7)).7
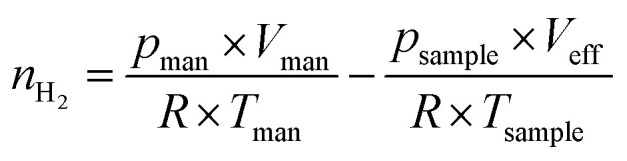
with *n*_H_2__ = amount of hydrogen (in mol) consumed during reduction, *p*_man_ = pressure in the manifold (in Pa), *V*_man_ = calibrated volume of the manifold (in m^3^), *R* = ideal gas constant (= 8.3145 J mol^−1^ K^−1^), *T*_man_ = temperature in the manifold (= 298 K), *p*_sample_ = pressure after hydrogen consumption in the reactor (in Pa), *V*_eff_ = effective volume considering the temperature gradient in the reactor at *T*_sample_ (in m^3^), *T*_sample_ = sample temperature (in K).

Since the reduction of 1 mol surface Cu_2_O with H_2_ yields 2 mol Cu^0^, the consumed molar amount of H_2_ is equal to twice the amount of accessible Cu surface atoms. Hence, the specific copper surface area SA_Cu_ and the dispersion *D*_Cu_ can be determined utilizing eqn (8) and (9), respectively.^54^8
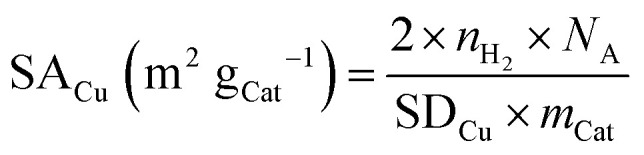
with SA_Cu_ = specific Cu surface area (in m^2^ g_Cat_^−1^), *N*_A_ = Avogadro's number (= 6.022 × 10^23^ mol^−1^), SD_Cu_ = atom surface density of Cu (= 1.46 × 10^19^ m^−2^),^52^*m*_Cat_ = mass of catalyst used in the analysis (in g).9
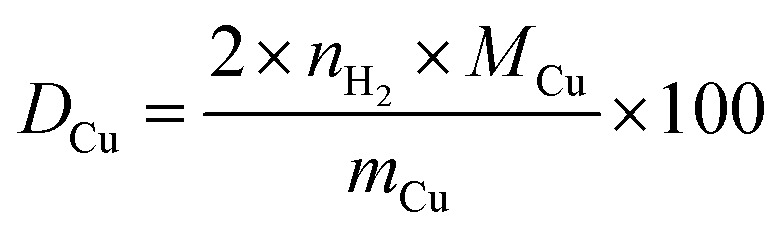
with *D*_Cu_ = dispersion of Cu (in %), *M*_Cu_ = molar mass of Cu (= 63.546 g mol^−1^), *m*_Cu_ = mass of Cu used in the analysis (in g).

The average particle diameter of Cu *d*_Cu_ can be estimated according to eqn (10).^55^ This model evaluation is based on the assumption of spherical particles embedded in the support. Hence, only half of their surface area is accessible.10
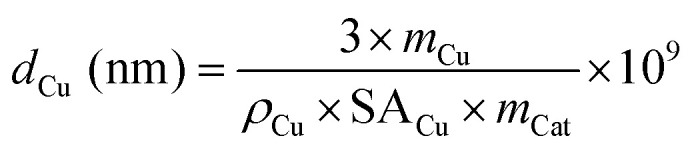
with *d*_Cu_ = average Cu particle diameter assuming embedded spherical particles (in nm), *ρ*_Cu_ = density of metallic Cu (= 8.94 × 10^6^ g m^−3^).^56^

### 2.7. Thermogravimetric analysis (TGA)

The copper loading of the catalysts was determined *via* oxidation–reduction–oxidation–reduction treatments in a NETZSCH STA 449F3 Jupiter TGA/DSC apparatus. To ensure complete oxidation, the samples were subjected to a temperature program consisting of heating with 5 °C min^−1^ from 25–400 °C, an isothermal period of 30 min and subsequent cooling to 25 °C with 5 °C min^−1^ in a gas atmosphere of 10 vol% O_2_ (99.999%, Linde) in Ar (99.999%, Linde) with a total flow rate of 100 ml min^−1^. The same temperature program was applied for reduction in a gas mixture of 10 vol% H_2_ (99.999%, Linde) in Ar with a total flow rate of 100 ml min^−1^. The reduction of the catalysts corresponds to the transformation of CuO to Cu^0^, where the weight loss of oxygen is quantified and can be utilised for the calculation of the copper loading. The additional oxidation and reduction steps were conducted to ensure the reproducibility of the measurements.

## 3. Results and discussion

**Fig. 1:**
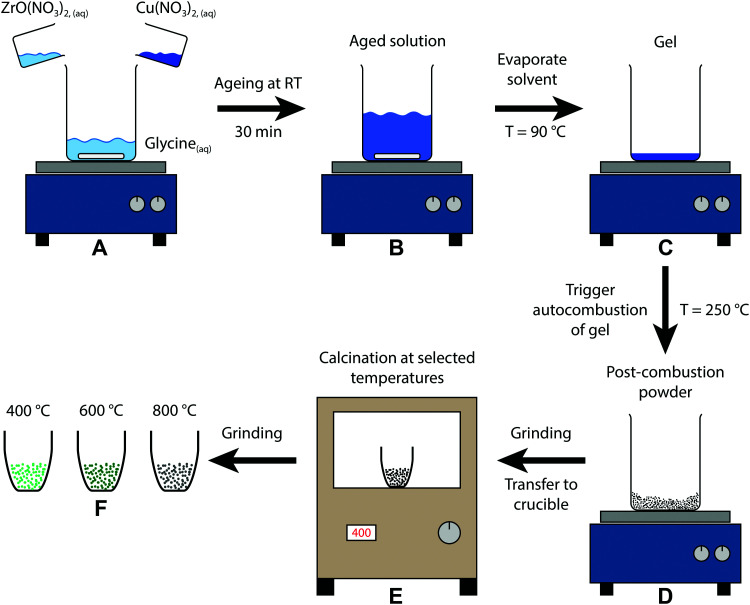
Schematic depiction of the sol–gel autocombustion yielding the three samples of this study by calcination of the post-combustion precursor at different temperatures.

A schematic representation of the sol–gel autocombustion approach is depicted in Fig. 1. The educts are dissolved separately in deionised water and then combined in one solution (A), where glycine acts as a complexing agent for copper and zirconium, ensuring a high dispersion in the dissolved state even upon removal of water.^33^ After ageing (B), the solvent is removed by evaporation at 90 °C, leading to the formation of a gel (C). The autocombustion is triggered by increasing the temperature to approximately 250 °C, causing a spontaneous strongly exothermic reaction of glycine and nitrate.^33^ The resulting powder (D) is still amorphous and can be calcined at selected temperatures (E), yielding the samples (F) in this study. This procedure can be conducted without copper as well, only utilizing ZrO(NO_3_)_2_ and glycine. The obtained Zr precursor exhibits a similar tunability of its structure by application of different calcination treatments, whereas the temperature regions of stability are distinct from the copper-containing samples.

Variation of the calcination temperature from 400–900 °C for the pure amorphous Zr precursor material reveals a dependency of the colour on the calcination temperature (see ESI,† Fig. S1). The colour changes from dark brown at 400 °C, over brown to white at 800 °C. A tentative explanation for the darker colour at lower calcination temperatures can be given by an elevated number of defects in ZrO_2_ acting as colour centres.^57,58^ At lower calcination temperatures (400–600 °C), more oxygen vacancies, which can be created by the reaction of carbonaceous species from the synthesis with lattice oxygen,^59^ can be retained. Stabilisation of the tetragonal structure at lower calcination temperatures (*cf.* Fig. S5, ESI†) is mainly ascribed to the presence of an elevated number of oxygen vacancies (which are themselves stabilised by the nanoparticle size effect).^59^ Since the calcination was conducted in air, the oxygen vacancies can be quenched at higher temperatures, resulting in monoclinic zirconia as the predominant polymorph. This is a result of the missing stabilisation of the tetragonal phase due to a decreased number of oxygen vacancies, which in turn could explain the white colour, as less colour centres in the form of oxygen vacancies are present. We have tried to assess the influence of surface-near defects by evaluating the Zr 3d spectra (Fig. S4, ESI†), but while we did observe a high number of sub-stoichiometric Zr oxides for all samples, a clear trend with respect to the annealing temperature was not obtained. We hence conclude that mostly bulk-related vacancies contribute to the colour. Note that the different sample colour after calcination can be masked by both carbon residues from the synthesis^60^ and the addition of copper. Additionally, copper may affect the stability of vacancies in ZrO_2_, altering the prerequisites altogether. One example of the effect of Cu addition in binary Cu/ZrO_2_ systems was provided by doping ZrO_2_ with copper in a co-precipitation synthesis, which leads to increasing stabilisation of the tetragonal and cubic ZrO_2_ polymorphs and a reduction of the crystallite size of the zirconia phase with increasing copper content.^61^ Another study of three binary Cu/ZrO_2_ catalysts for methanol synthesis prepared by impregnation and co-precipitation techniques reported that the stabilisation of the tetragonal polymorph at lower temperature is caused by the presence of oxygen vacancies. Furthermore, incorporation of Cu^+^ or Cu^2+^ into the ZrO_2_ lattice compensates for the negative charge of vacancies in ZrO_2_, further contributing to the stabilisation of the tetragonal polymorph.^62^

The three Cu/ZrO_2_ samples in this study exhibit a distinct colour after calcination, which is clearly different from the colour of the black post-combustion powder (see ESI,† Fig. S2). The sample calcined at 400 °C for 2 h in air (termed CZ400) is between green and turquoise, treatment at 600 °C for 2 h (sample termed CtZ600) leads to a darker green colour and calcination at 800 °C for 2 h (sample termed CmZ800) yields a grey powder. A list of the investigated Cu/ZrO_2_ samples is provided in Table 1. The green colour of CZ400 indicates the incorporation of Cu into ZrO_2_, which was also observed by Tada *et al.*^63,64^ upon impregnating amorphous zirconia with copper nitrate, identifying the resulting phase with X-ray absorption studies as an amorphous ternary Cu_*x*_Zr_*y*_O_*z*_ compound. The grey colour of CmZ800 can be explained as a mixture of the purely white m-ZrO_2_ and the black CuO, whereas the dark green colour of CtZ600 might be interpreted as an intermediate state, consisting of partially remaining Cu_*x*_Zr_*y*_O_*z*_ as well as already present CuO and t-ZrO_2_.

**Table 1:** Sol–gel autocombustion-synthesised Cu/ZrO_2_ samples including the calcination temperature, the support polymorph and the copper loading determined by TGA

Acronym	Calcination temperature/°C	ZrO_2_ polymorph after calcination	Copper loading from TGA (reduced state)/wt%
CZ400	400	Amorphous/tetragonal	6
CtZ600	600	Tetragonal	6
CmZ800	800	Monoclinic	6

The copper loading of the samples was determined *via* an oxidation–reduction–oxidation–reduction cycle in a thermogravimetric analysis (TGA) setup as described in Section 2.7. The mass decrease in the reduction with H_2_ up to 400 °C corresponds to the Cu loading, which is identical for all three samples. This confirms the expectations, as all catalysts were obtained by calcination of the same post-combustion material.

The effect of the different calcination treatments on the structure of the pure ZrO_2_ samples is visualised in *ex situ* XRD measurements depicted in the ESI† (Fig. S5). At 400 °C, the material remains amorphous, whereas at 500 °C, pure t-ZrO_2_ is formed. From 600 °C to 900 °C, the amount of m-ZrO_2_ increases until only small contributions of t-ZrO_2_ prevail. Similar trends can be observed in an *in situ* XRD experiment with the post-combustion powder of ZrO_2_ (ESI,† Fig. S6). Heating this precursor in 20 vol% O_2_ in Ar from 25–800 °C reveals an initial reordering to amorphous zirconia starting around 200 °C. At 460 °C, the tetragonal phase starts to form until the evolution of m-ZrO_2_ is observable at approximately 700 °C. The two separate approaches employing either isothermal calcination (characterised by *ex situ* XRD in the ESI† in Fig. S5) or a heating ramp in the *in situ* XRD experiments (ESI,† Fig. S6) lead to apparently different temperature stability regions of the polymorphs, which is a consequence of the different time the samples are exposed to a certain temperature. This is visible in the appearance of m-ZrO_2_ already at 600 °C following isothermal calcination for 2 h, while it is formed at 700 °C in the *in situ* XRD characterisation.

The Cu/ZrO_2_ samples display similar trends, but the effect of copper on the stability of the zirconia polymorph is clearly visible. After isothermal calcination at 400 °C for 2 h in air, small amounts of t-ZrO_2_ are already present in the sample (see *ex situ* XRD characterisation in the ESI† in Fig. S7), next to a mostly amorphous state. At 600 °C, pure tetragonal zirconia is obtained, whereas calcination at 800 °C yields pure m-ZrO_2_. Comparison to the isothermal calcination treatments of pure ZrO_2_ shows that the stability region of t-ZrO_2_ is expanded by the addition of copper, but the transformation kinetics are also accelerated. This means that t-ZrO_2_ is stabilised in the presence of Cu at an extended temperature range, but the transformation to m-ZrO_2_ occurs faster at high temperatures as well, as compared to pure ZrO_2_.

**Fig. 2:**
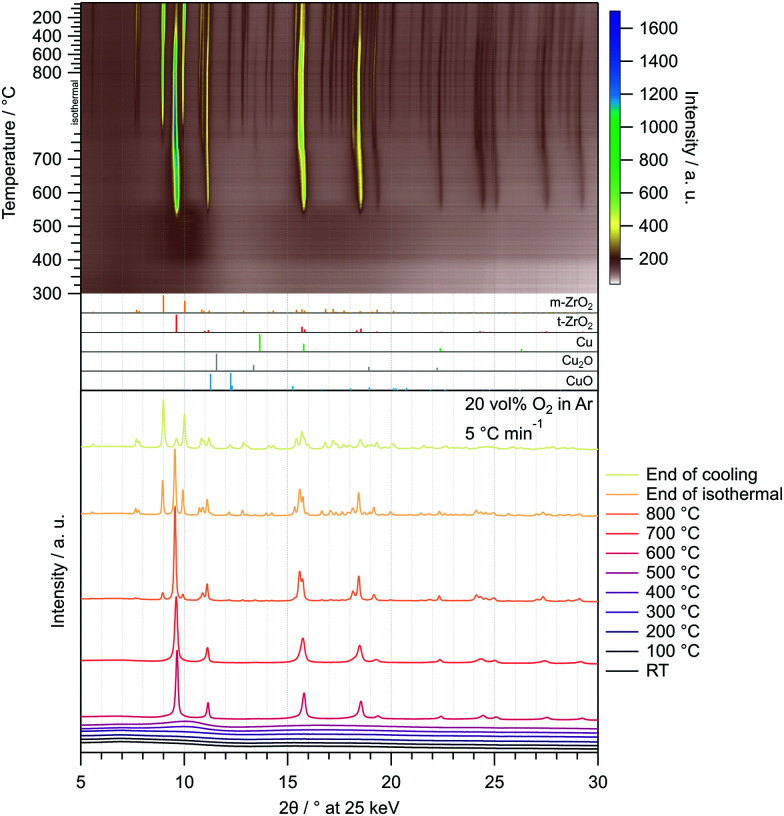
Temperature-resolved *in situ* XRD investigation of the Cu/ZrO_2_ precursor after combustion by calcination in 20 vol% O_2_ in Ar with a heating rate of 5 °C min^−1^ between 25 °C and 800 °C. In the uppermost panel, the entire treatment consisting of heating to 800 °C (depicted from 300 °C), an isothermal period of 30 min and cooling to 25 °C with 20 °C min^−1^ is illustrated as a heat map. Selected diffractograms are depicted in the bottom panel. The patterns were recorded utilizing synchrotron radiation at 25 keV (*λ* = 0.4959 Å). References were taken from the ICDD database.^65–70^

The corresponding *in situ* XRD calcination treatment of the amorphous Cu/ZrO_2_ post-combustion powder in 20 vol% O_2_ in Ar from 25–800 °C (Fig. 2) reveals that the onset temperature of t-ZrO_2_ formation is higher than in pure ZrO_2_ (≈510 °C *vs.* ≈460 °C). The same is true for the evolution of m-ZrO_2_ (≈770 °C in Cu/ZrO_2_*vs.* ≈700 °C in ZrO_2_).

No copper phase is detected in the XRD pattern for the samples CZ400 and CtZ600 after isothermal calcination (ESI,† Fig. S7 at 400 °C and 600 °C, respectively), whereas CuO is clearly observable for CmZ800. This further supports the formation of an amorphous Cu_*x*_Zr_*y*_O_*z*_ phase as proposed by Tada *et al.*^63,64^ After MSR, metallic copper is found in all samples, but only in trace amounts in CZ400 (ESI,† Fig. S7).

The specific surface area of the Cu/ZrO_2_ catalysts was characterised by N_2_ adsorption according to the BET method as well as dissociative N_2_O adsorption followed by H_2_ titration (Table 2). CZ400 exhibits the highest BET surface area as it was treated at the lowest temperature, whereas CmZ800 has the lowest value. This trend is not directly reflected in the specific copper surface area. Two cycles of dissociative N_2_O chemisorption were conducted on each sample, where each cycle consists of pre-reduction at 300 °C in pure H_2_ to convert Cu quantitatively into the metallic state, selective surface oxidation with N_2_O at 70 °C and reduction of the formed surface Cu_2_O layer with H_2_ at 300 °C. The highest Cu surface area is observed for CtZ600, followed by CZ400 and finally CmZ800 with the lowest value. An analogous trend was reported by Wang *et al.*^30^ with Cu/ZrO_2_ catalysts prepared by oxalate gel-coprecipitation, where the precursor was calcined at different temperatures from 350–750 °C. Based on dissociative N_2_O adsorption studies, they observed the highest specific copper surface area for the sample treated at the intermediate temperature of 550 °C and interpreted this in terms of agglomeration of copper or metal–support interaction in Cu–ZrO_2_.^30^ An alternative tentative explanation could be a very high stability of the Cu_*x*_Zr_*y*_O_*z*_ in CZ400 in reducing atmosphere, indicating that most of the Cu remains inaccessible in the bulk of the oxide. To circumvent this deficiency, XPS in combination with the BET surface area is utilised to calculate the specific copper surface area (ESI† in Section S3). The comparison of the obtained SA_Cu_(XPS) and SA_Cu_(N_2_O) confirms the magnitude of the specific copper surface area, but the trend of SA_Cu_(XPS) follows the BET surface area, where CZ400 exhibits the largest and CmZ800 the lowest value.

**Table 2:** BET surface area, specific Cu surface area, Cu dispersion and average Cu particle size of the copper-containing catalysts determined by dissociative N_2_O adsorption

Acronym	BET surface area/m^2^ g_Cat_^−1^	Specific Cu surface area/m^2^ g_Cat_^−1^	Cu dispersion/%	Average Cu particle size/nm
CZ400	32	1.9;^a^ 1.4^b^	4.9,^a^ 3.5^b^	11,^a^ 15^b^
CtZ600	7	7.6;^a^ 4.5^b^	20,^a^ 12^b^	3,^a^ 4^b^
CmZ800	2	0.9;^a^ 0.4^b^	2.2,^a^ 1.1^b^	23,^a^ 45^b^

a First cycle.

b Second cycle on the same sample.

In the second cycle, the copper surface area is decreased in all samples, which can be attributed to sintering of metallic Cu under reductive atmosphere. The extent of sintering matches the trend of the calcination temperature. While the decrease of the Cu surface area amounts to approximately 30% in CZ400, it increases to 40% for CtZ600 and 50% for CmZ800. This implies that CZ400 is the catalysts that is most resistant to deactivation by sintering under the applied conditions.

**Fig. 3:**
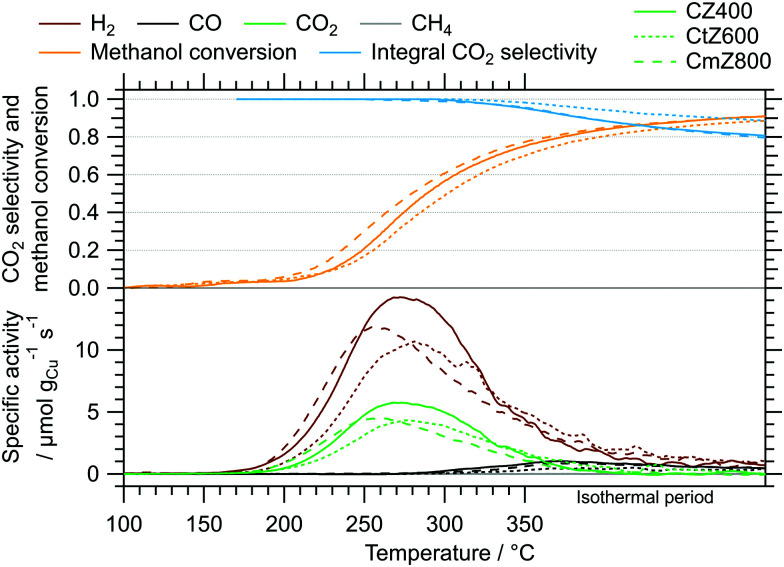
MSR profiles of CZ400, CtZ600 and CmZ800 between 100 °C and 350 °C including an isothermal period of 30 min. Colour code: orange – methanol conversion, blue – integral CO_2_ selectivity, specific activity of brown: H_2_, black: CO, green: CO_2_, grey: CH_4_. Heating rate: 5 °C min^−1^; sample mass: CZ400 – 18.6 mg, CtZ600 – 21.6 mg, CmZ800 – 20.2 mg.

As a measure of the MSR performance of pure ZrO_2_ prepared *via* the sol–gel autocombustion, the mostly amorphous ZrO_2_ obtained by calcination in air at 400 °C for 2 h (termed Z400) was selected as a representative sample. The MSR profiles of Z400 measured between 100 °C and 350 °C in the recirculating batch reactor are depicted in the ESI† in Fig. S8. Two cycles were conducted, one without pre-treatments and the other one including pre-oxidation and pre-reduction. The onset temperatures of all major products – H_2_, CO, CO_2_ and CH_4_ – are located at approximately 300 °C. Additionally, we observe formation of formic acid at around 310 °C. We explain the formation of methane and formic acid on Z400 by different reaction mechanisms occurring with and without Cu. The formation of formic acid on Z400 starts around 310 °C, indicating that these species are tightly bound to the catalyst and persist conversion to CO_2_ on pure ZrO_2_. The presence of Cu facilitates the conversion of formate species (as the precursor of formic acid) to CO_2_, which is evident by the low CO_2_ onset temperature of ≈150 °C in the Cu-containing catalysts (Fig. 3). We observe the formation of methane at ≈300 °C on Z400, whereby the formation of methane is suppressed for the Cu-containing systems. This is due to the fast conversion of the precursors for methane formation to CO_2_ and H_2_ before the mechanism of methane formation plays a significant role. Additionally, we cannot exclude that the reactivity of centres active for methane formation in pure ZrO_2_ are altered or suppressed by the addition of Cu.^19^

The MSR profiles obtained on CZ400, CtZ600 and CmZ800 in the batch reactor are summarized in Fig. 3 and Table 3. In comparison to Z400, these three catalysts exhibit a drastically different behaviour in MSR. The total formation rates of both cycles of Z400 are two orders of magnitude lower than for these copper-containing catalysts. The onset temperatures of H_2_ (≈150 °C), CO (≈270 °C) and CO_2_ (≈150 °C) are almost identical for all three samples, as is the general progression of the specific activity, CO_2_ selectivity and methanol conversion. This implies that the crystal structure of ZrO_2_, which varies from amorphous over tetragonal to monoclinic in these catalysts, does not significantly impact the selectivity patterns of these systems in MSR. Additionally, the formation of methane and formic acid observed on Z400 (ESI,† Fig. S8 and S9) is absent for CZ400, CtZ600 and CmZ800. In case Cu is present, the onset temperature of CO formation is shifted to lower temperatures (270 °C *vs.* 300 °C), as well as the higher specific activity towards CO. We interpret this as Cu-enhanced kinetics of the support, yielding more CO already at lower temperatures. Note that the decrease of the CO_2_ selectivity at higher temperatures in Fig. 3 is characteristic for MSR operation in a recirculating batch reactor. As we provide no constant feed of methanol and water in batch reactor measurements, methanol is successively depleted and the corresponding formation rates of H_2_ and CO_2_ decrease, while the reverse water–gas shift reaction, converting H_2_ and CO_2_ to CO and water, increasingly occurs. Therefore, the decrease in the CO_2_ selectivity is mechanistically connected to the transition from MSR to the reverse water–gas shift reaction in a batch reactor and should not be interpreted as a purely MSR-specific value above methanol conversions of ≈40%.

**Table 3:** Summary of MSR key parameters of the samples in the batch reactor

	CZ400	CtZ600	CmZ800
Onset *T*(H_2_)/°C	150	150	150
Onset *T*(CO)/°C	270	270	270
Onset *T*(CO_2_)/°C	150	150	150
*T*(<95%)/°C	150–350 (start of isothermal)	150–350 (8 min isothermal)	150–350 (1 min isothermal)
Max. activity CO_2_/μmol g_Cu_^−1^ s^−1^	5.8	4.3	4.5
	270 °C	280 °C	260 °C
Max. activity H_2_/μmol g_Cu_^−1^ s^−1^	14.3	10.7	11.9
	270 °C	280 °C	255 °C
*E*_A_(CO_2_)/kJ mol^−1^	78	77	78

**Fig. 4:**
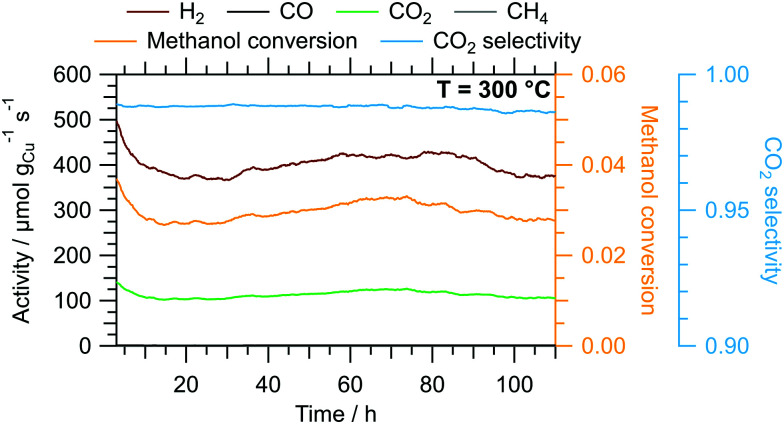
Isothermal long-term MSR test of CZ400 at 300 °C in a continuous flow reactor with a GHSV of 1925 h^−1^. Colour code: orange – methanol conversion, blue – CO_2_ selectivity, specific activity of brown: H_2_, black: CO, green: CO_2_, grey: CH_4_. Sample mass: 40.0 mg. We corrected spikes caused by fluctuations in the supply of the initial MSR mixture during the experiment assuming a linear trend between the adjacent data points as well as a similar noise level.

The results of the isothermal long-term characterisation of CZ400 in MSR at 300 °C is depicted in Fig. 4. After a slight initial decrease of the specific activity by approximately 25% in the first 15 h on stream, the deactivation trend wanes and the performance remains stable up to 110 h time on stream. The CO_2_ selectivity remains high at ≈99% throughout the experiment. The comparably low methanol conversion is a result of the small accessible specific copper surface area (Table 2) in combination with the low sample amount.

The isothermal MSR flow characterisation of CtZ600 and CmZ800 are visualised in the ESI† in Fig. S10 and S11. Both exhibit a much stronger deactivation trend that continues throughout the entire experiment. The specific activity of CtZ600 decreases by about 90% and of CmZ800 by approximately 80% during 100 h time on stream. In combination with the initially already lower activity of these catalysts, the CO_2_ selectivity could not be determined reliably after 20 h time on stream, because the CO formation dropped below the detection limit of the GC (20 ppm). These results clearly show that despite the apparent indifference of the ZrO_2_ polymorph on the CO_2_ selectivity, the activity as well as the long-term stability are significantly improved by the presence of the amorphous ZrO_2_ support. The general trends of the deactivation, which can most likely be ascribed to sintering of metallic copper, have already been observed in the dissociative N_2_O adsorption experiments, where the decrease in specific copper surface area was smaller in CZ400 than in CtZ600 and CmZ800 (see Table 2), although the conditions were different (MSR mixture *vs.* reduction in H_2_).

**Fig. 5:**
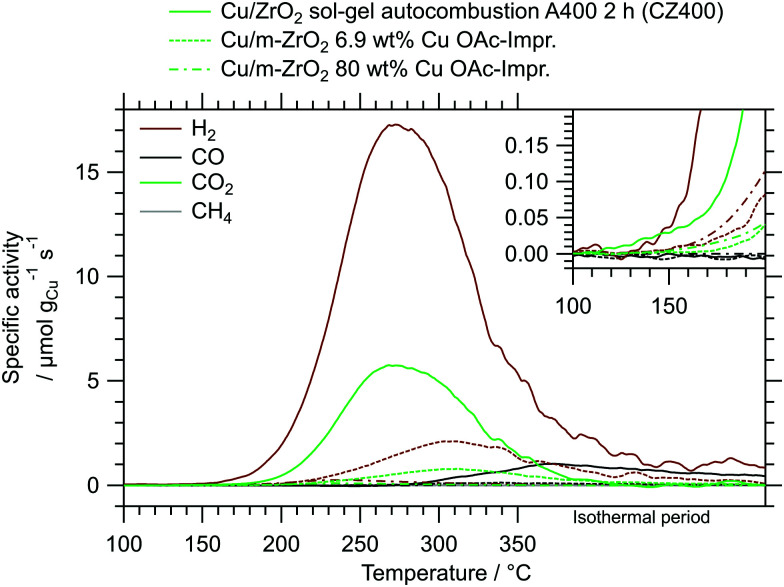
Comparison of CZ400 with other impregnated Cu/ZrO_2_ catalysts, one from literature^19^ and another analogous system with a higher Cu loading, measured in the same recirculating batch reactor setup under identical MSR conditions.

**Fig. 6:**
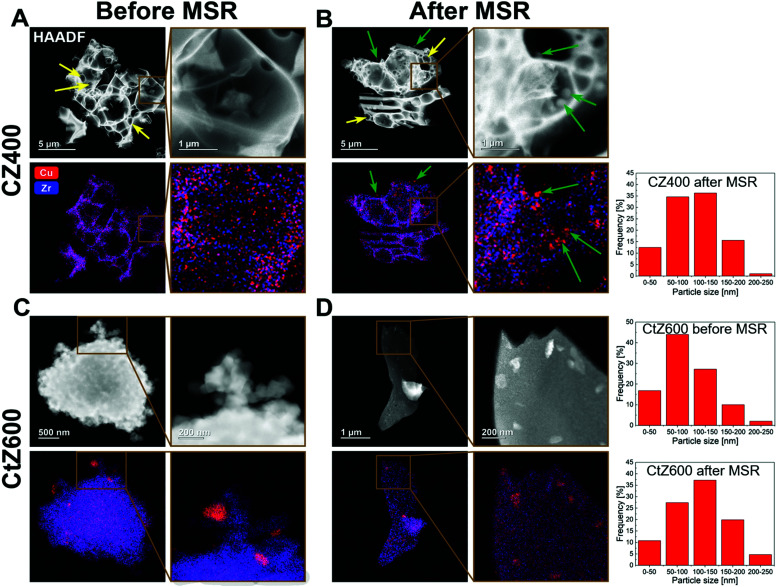
HAADF-STEM images and EDX maps of the samples CZ400 and CtZ600 after calcination and after one MSR cycle in the recirculating batch reactor. For each CZ400 (panels A and B) and CtZ600 (panels C and D) state, one image including an enlarged region is depicted (small brown frames indicate the selected area), each with one HAADF image and the corresponding EDX map of Cu in red and Zr in blue. In panels A and B, combustion pores are indicated with yellow arrows. In panel B, Cu particles observed after MSR are marked with green arrows, where the same particles are highlighted in the respective HAADF-STEM images and EDX maps. Panel A: CZ400 after calcination; panel B: CZ400 after MSR350; panel C: CtZ600 after calcination; panel D: CtZ600 after MSR350. In the right panels, we show the exemplary particle size histograms based on the TEM/EDX analysis for two representative catalysts CZ400 and Ct600.

The beneficial properties of CZ400 in terms of specific activity are especially pronounced in a comparison with analogous systems from literature. In Fig. 5, the MSR performance of CZ400 is related to the best performing impregnated Cu/ZrO_2_ catalysts from our workgroup,^19^ since they were characterised under identical catalytic conditions in the same batch reactor setup. This guarantees optimal comparability and illustrates the remarkable specific activity of CZ400, which exhibits a maximum specific activity towards H_2_ that is approximately 8 times higher than the Cu/m-ZrO_2_ system prepared by aqueous impregnation with a copper loading of 6.9 wt% and 66 times higher than the analogously synthesised Cu/m-ZrO_2_ catalyst with 80 wt% Cu. Furthermore, a comparison of all five catalysts (CZ400, CtZ600, CmZ800 and the two abovementioned catalysts) in terms of their turnover frequency (TOF) as well as the specific activity in μmol g_Cat_^−1^ s^−1^ can be found in the ESI† in Fig. S12 and S13, respectively. The TOF values of CZ400 are comparable with the Cu/m-ZrO_2_ catalyst prepared by aqueous impregnation with a loading of 6.9 wt% Cu described in ref. 19, where its performance is compared to other systems from literature. The TOF values were not used in the manuscript, because the catalysts are altered significantly upon MSR (*cf.*Fig. 6). This change caused by exposure to the MSR mixture, however, is not represented in the measurements for the determination of the specific copper surface area, as they were conducted prior to MSR.

To identify the reason for the different deactivation behaviour, combined HAADF-STEM and EDX investigations were performed on CZ400 as the most sinter-resistant sample and CtZ600 as the catalyst exhibiting the strongest deactivation. The images are depicted in Fig. 6 and provide a direct comparison of the Cu and ZrO_2_ morphology and elemental distribution after calcination and after one MSR cycle in the recirculating batch reactor. In Panel A, CZ400 after calcination exhibits a homogeneous distribution of Cu and Zr, with merely sporadic large Cu particles (around 120 nm) being visible. Additionally, this sample exhibits the typical combustion pores.^42,44^ After MSR (Panel B), an increased number of particles with sizes of up to 200 nm can be found, which are primarily located in the pores. The porous morphology is retained and regions with less agglomeration of Cu can also be observed.

In Fig. 6 Panel C, CtZ600 exhibits a completely different morphology without clearly visible pores. In contrast to CZ400, larger Cu particles of up to 200 nm are visible after calcination in CtZ600. After MSR, these agglomerates become more frequent and the general morphology of the catalyst changes from an apparently loose network of particles to continuous large platelets of zirconia with copper particles on top. This drastic change of the morphology of CtZ600 in combination with the increased frequency of Cu agglomerates could serve as an explanation for the much more severe deactivation of this catalyst, as compared to CZ400. The latter mostly retains its initial morphology, but also shows more Cu particles accounting for the slight initial decrease of the activity in MSR.

The sintering stability argument is further strengthened by the particle size histograms provided for the most active (CZ400) and one deactivating catalyst (CtZ600) before and after MSR operation (note that for CZ400, no such particle size histogram could be reliably provided before MSR, as we assume that for CZ400 a ternary mixed Cu–Zr–O oxide is prevalent before catalysis). However, after MSR operation the amount of smaller particles is clearly higher for CZ400 and for CtZ600, we find an increased amount of significantly larger particles.

## 4. Conclusions

The adaption of an established synthesis approach of the sol–gel autocombustion to an alternative class of Cu–ZrO_2_ catalysts enables the tuning of the ZrO_2_ polymorph and the interface between Cu and ZrO_2_*via* the calcination temperature. This creates the possibility to prepare samples with very similar chemical properties arising from identical precursors, which represents one of the key parameters governing the CO_2_ selectivity in MSR.^19^ Altering the morphology of the catalysts allows to optimise the activity and long-term stability. Employing this approach, a highly CO_2_-selective, active and long-term stable Cu/ZrO_2_ catalyst for MSR was synthesised, which outperforms various impregnated and co-precipitated samples from literature in terms of both specific and long-term stable activity.

The advantages of the sol–gel autocombustion were clearly demonstrated by exploiting the typical formation of combustion pores,^44^ creating a unique catalyst morphology. Upon calcination of the post-combustion precursor at 400 °C (yielding CZ400), this special porous structure stemming from the combustion is still retained and serves as an explanation for its increased resistance towards deactivation by hindering the diffusion of Cu at the surface and sintering. This morphology is lost upon calcination at higher temperatures and, hence, CtZ600 and CmZ800 exhibit increased deactivation through sintering.

## Conflicts of interest

There are no conflicts to declare.

## Supplementary Material

QM-005-D1QM00641J-s001
