# Supplementary material for: The sol–gel autocombustion as a route towards highly CO2-selective, active and long-term stable Cu/ZrO2 methanol steam reforming catalysts
Source: Mater Chem Front. 2021 May 20;5(13):5093–105. doi: 10.1039/d1qm00641j (PMC8238116; doi:10.1039/d1qm00641j)
Supplement: QM-005-D1QM00641J-s001 [file QM-005-D1QM00641J-s001.pdf]

Electronic Supplementary Material (ESI) for Materials Chemistry Frontiers.
This journal is © the Partner Organisations 2021

Supporting Information

The sol-gel autocombustion as a route towards highly CO_2-selective, active and long-term stable Cu/ZrO_2 methanol steam reforming catalysts

Kevin Ploner[1], Parastoo Delir Kheyrollahi Nezhad[1], Albert Gili[2,3], Franz Kamutzki[2], Aleksander Gurlo[2], Andrew Doran[4], Pengfei Cao[5], Marc Heggen[5], Nicolas Köwitsch[6], Marc Armbrüster[6], Maximilian Watschinger[1], Bernhard Klötzer[1] and Simon Penner[1,*]

[1]Department of Physical Chemistry, University of Innsbruck, Innrain 52c, A-6020 Innsbruck

[2]Chair of Advanced Ceramic Materials, Institut für Werkstoffwissenschaften und -technologien, Technical University Berlin, Hardenbergstr. 40, D-10623 Berlin, Germany

[3]Institute of Chemistry, Technical University Berlin, Sekretariat TC 8, Straße des 17. Juni 124, D-10623 Berlin, Germany

[4]Advanced Light Source, Lawrence Berkeley National Laboratory, Berkeley, California 94720, USA

[5]Ernst Ruska-Centre for Microscopy and Spectroscopy with Electrons, Forschungszentrum Jülich GmbH, Leo-Brandt-Str. 1, D-52428 Jülich, Germany

[6]Faculty of Natural Sciences, Institute of Chemistry, Materials for Innovative Energy Concepts, Technical University Chemnitz, Straße der Nationen 62, D-09111 Chemnitz, Germany

Keywords: amorphous zirconia, tetragonal zirconia, monoclinic zirconia, copper, catalyst morphology

*Corresponding Author: simon.penner@uibk.ac.at, Tel: 004351250758003

1. Detailed experimental

1.1. *Ex situ* X-ray diffraction

The *ex situ* powder X-ray diffraction experiments were conducted on a Stadi P diffractometer (STOE & Cie GmbH, Darmstadt, Germany) in transmission geometry equipped with a MYTHEN2 DCS6 detector system (DECTRIS Ltd., Switzerland). A Mo X-ray tube (GE Sensing & Inspection Technologies GmbH, Ahrensburg, Germany) operated with 40 mA and 50 kV in combination with a curved Ge(111) monochromator crystal provided the Mo-$K_{\alpha 1}$ radiation with a wavelength of 0.7093 Å. The evaluation of the diffractograms was performed using the $WinX^{POW}$ software[1] and phase assignment was accomplished with references retrieved from the ICDD database[2].

1.2. Calculation of the turnover frequency

The turnover frequency (TOF) can be calculated *via* differentiation of the partial pressures with respect to the time and consecutive application of the ideal gas law, yielding the rates in molar amounts per time. Further multiplication with Avogadro's number and normalisation to the number of accessible active sites yields the TOF in s^{-1}. Since the nature of the active site in Cu-based MSR catalysts is still under debate[3], the number of accessible copper surface atoms is assumed to be a reasonable estimate for this parameter. This value is computed utilizing the results of the dissociative N_2O adsorption, which provides the specific copper surface area. Assuming an equal distribution of the Cu(100), Cu(110) and Cu(111) surface planes, Evans *et al.*[4] supply the surface atom density of copper ($= 1.46 \cdot 10^{19}\ m^{-2}$), thus, allowing the estimation of the TOF (see Equation S1).

$$TOF\ (s^{-1}) = \frac{r * V * N_A}{R * T * SA_{Cu} * SD_{Cu} * m_{Cat}} \qquad \text{Equation S1}$$

with TOF = turnover frequency (in s^{-1}), r = formation rate of a product (in Pa s^{-1}), V = volume of the reactor (= 13.8 · 10^{-6} m^3), N_A = Avogadro's number (= 6.022 · 10^{23} mol^{-1}), R = ideal gas constant (= 8.3145 J mol^{-1} K^{-1}), T = temperature in the reactor (= 373 K, as other temperature-induced effects are considered via the Ar correction), SA_{Cu} = specific Cu surface area (in m^2 g_{Cat}^{-1}), SD_{Cu} = atom surface density of Cu (= 1.46 · 10^{19} m^{-2})[4], m_{Cat} = mass of catalyst used in the analysis (in g).

1.3. X-ray photoelectron spectroscopy (XPS)

The XPS experiments were conducted with a Thermo Scientific MultiLab 2000 spectrometer equipped with a monochromatic Al K_α X-ray gun and an Alpha 110 hemispherical sector analyser. The base pressure is kept in the low 10^{-9} mbar range and electrons with a kinetic energy of 6 eV are applied to the sample by a flood gun for charge compensation.

For quantitative determination of the Cu/Zr ratio, high-resolution spectra of the Zr 3d and the Cu $2p_{3/2}$ regions with a pass energy of 20 eV and an energy step size of 0.05 eV are recorded. A Shirley-type function is employed for description of the background. In addition to the relative sensitivity factors (RSFs)[5], the predictive G1 formula according to Gries[6] was utilised to account for different inelastic mean free paths for quantification. For qualitative comparison with the measured spectra, various Cu species were measured as reference samples using the same instrument, including metallic Cu (sputter-cleaned Cu foil, Goodfellow, ≥ 99.99%), Cu_2O (Sigma-Aldrich, anhydrous, ≥ 99.99% trace metals basis), CuO (Sigma-Aldrich, 99.999% trace metals basis) and $Cu(OH)_2$ (synthesized by precipitation with $CuSO_4 \cdot 5\ H_2O$ (Merck, for analysis, 99.7%) and NaOH (Roth, ≥ 99%)).

2. Images of the investigated samples

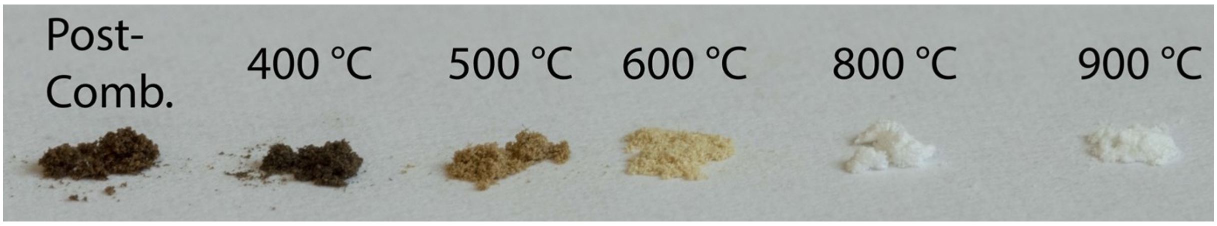

Figure S1: Picture of the pure ZrO_2 precursor after combustion and after different calcination treatments.

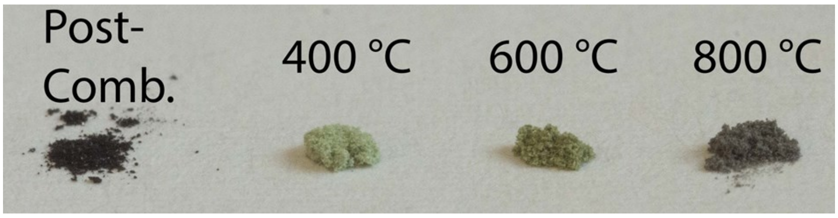

Figure S2: Picture of the Cu/ZrO_2 precursor with 10 mol% Cu after combustion and after calcination at different temperatures.

3. Confirmation of the specific copper surface area with X-ray photoelectron spectroscopy (XPS)

Based on these measurements, the atomic Cu/Zr surface ratio after calcination was determined (see Table S1). From these values, the Cu surface fraction can be calculated and multiplied by the specific BET surface area to receive an estimate of the specific Cu surface area, which is compared with the values obtained with dissociative N_2O adsorption in Table S1. SA_{Cu}(XPS) confirms the magnitude of $SA_{Cu}(N_2O)$, but also reflects the trend of the BET surface area, where CZ400 exhibits the highest value. Additionally, the determination of the Cu/Zr ratio with XPS is most reliable in a homogeneous sample, which is not given as soon as particles are formed. Therefore, the SA_{Cu}(XPS) would be underestimated in CtZ600 and CmZ800, where particle formation is visible with TEM. Furthermore, the surface Cu/Zr ratio increases in all samples after four MSR cycles, confirming that the MSR mixture is more efficient in extracting Cu from the $Cu_xZr_yO_z$ phase than mere reductive treatments in H_2. Since the BET surface area might change upon MSR, the SA_{Cu}(XPS) after catalysis are not calculated.

Table S1: Comparison of SA_{Cu} obtained with dissociative N_2O adsorption or XPS in combination with the BET surface area.

	CZ400	CtZ600	CmZ800
BET surface area / $m^2\ g_{Cat}^{-1}$	32	7	2
$SA_{Cu}(N_2O)$ / $m^2\ g_{Cat}^{-1}$	1.9	7.6	0.86
Atomic Cu/Zr surface ratio from XPS after calcination	0.19	0.23	0.29
SA_{Cu}(XPS) after calcination / $m^2\ g_{Cat}^{-1}$	5.2	1.3	0.45
Atomic Cu/Zr surface ratio from XPS after MSR	0.65	0.38	0.79

The XP spectra of the Cu 2p and Cu LMM regions of the samples are depicted in Figure S3 and the corresponding spectra of the Zr 3d region in Figure S4 panel A with the fits of the latter being visualised in Figure S4 panel B.

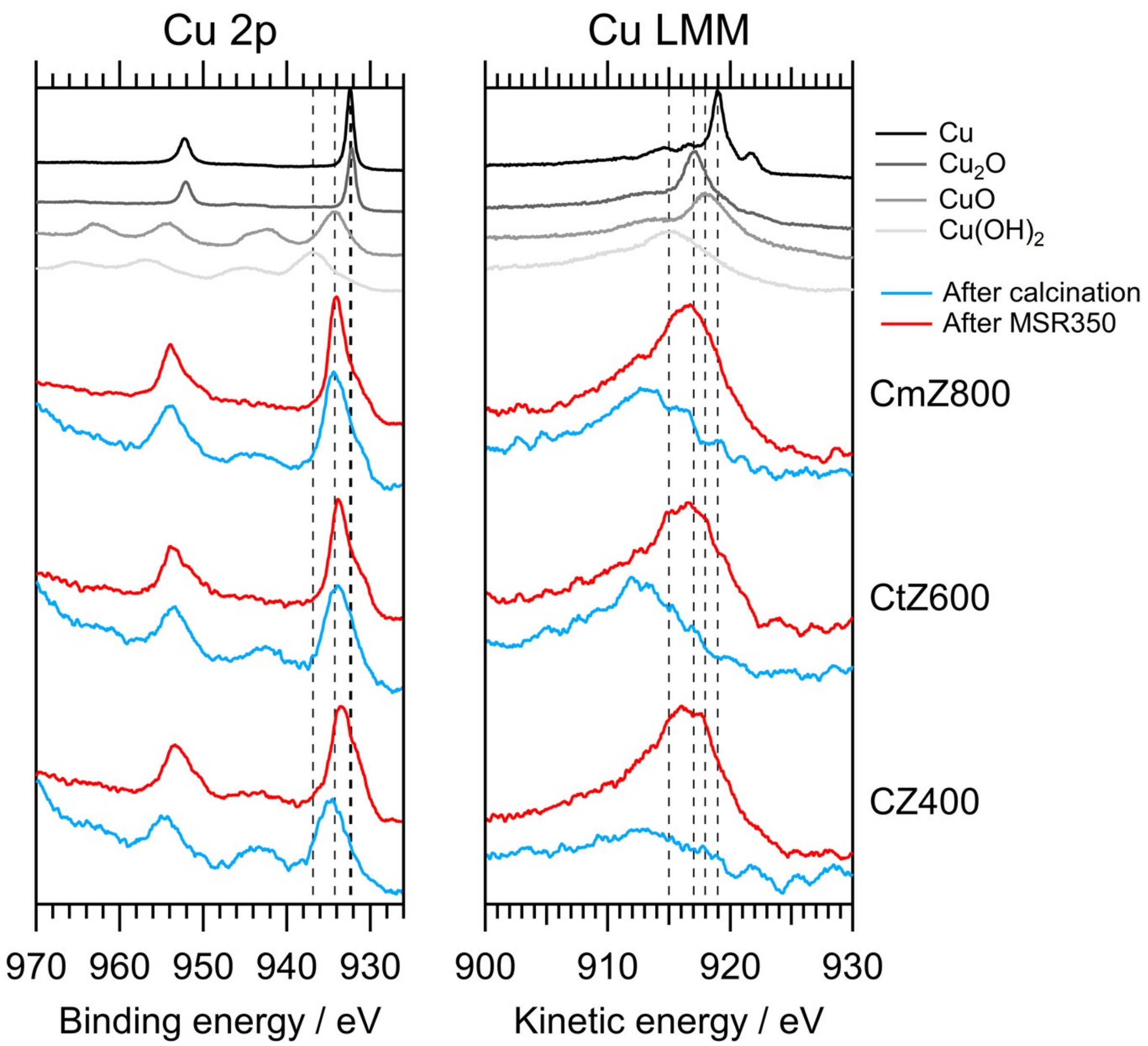

Figure S3: Cu 2p and Cu LMM regions of *ex situ* collected XP spectra of CZ400, CtZ600 and CmZ800 after calcination and after four MSR cycles. Reference spectra of Cu, Cu_2O, CuO and $Cu(OH)_2$ are visualized at the top. The sample spectra were smoothed to enhance the visibility of characteristic features.

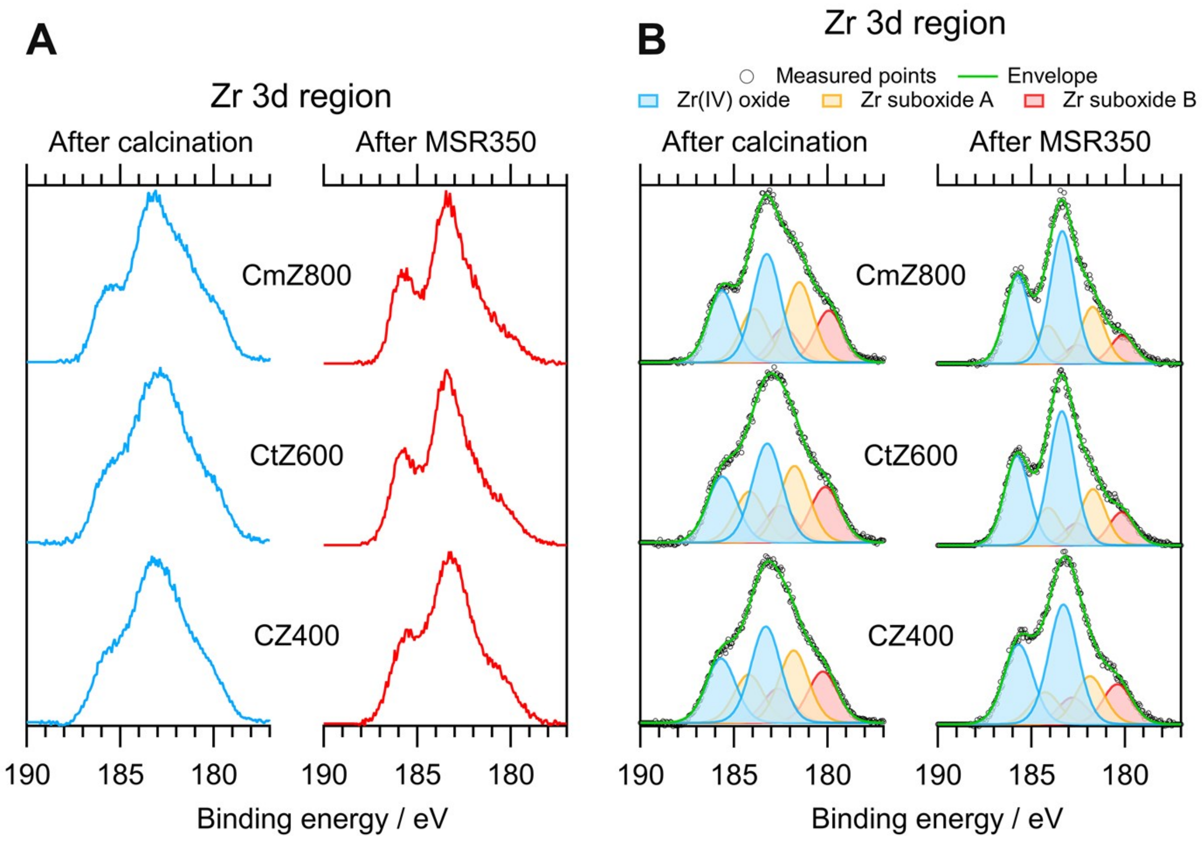

Figure S4: Panel A: Zr 3d region of *ex situ* collected XP spectra of CZ400, CtZ600 and CmZ800 after calcination and after four MSR cycles. Panel B: Fits of the same spectra in the Zr 3d region with the following constraints: Same full width at half maximum (FWHM) for all components; spin-orbit splitting of $3d_{5/2}$ and $3d_{3/2}$ of 2.4 eV for each species according to Nishino *et al.*[7]; area ratio of $3d_{5/2}$:$3d_{3/2}$ = 3:2 for each species.

Table S2: Surface concentration of Zr species after calcination and after four MSR cycles according to the fits in Figure S4 panel B.

Sample	Surface concentration after calcination / at%			Surface concentration after MSR / at%		
	Zr(IV) oxide	Zr suboxide A	Zr suboxide B	Zr(IV) oxide	Zr suboxide A	Zr suboxide B
CZ400	44	33	23	58	23	19
CtZ600	43	33	24	60	25	15
CmZ800	45	34	21	61	26	13

4. ***Ex situ* and *in situ* X-ray diffraction**

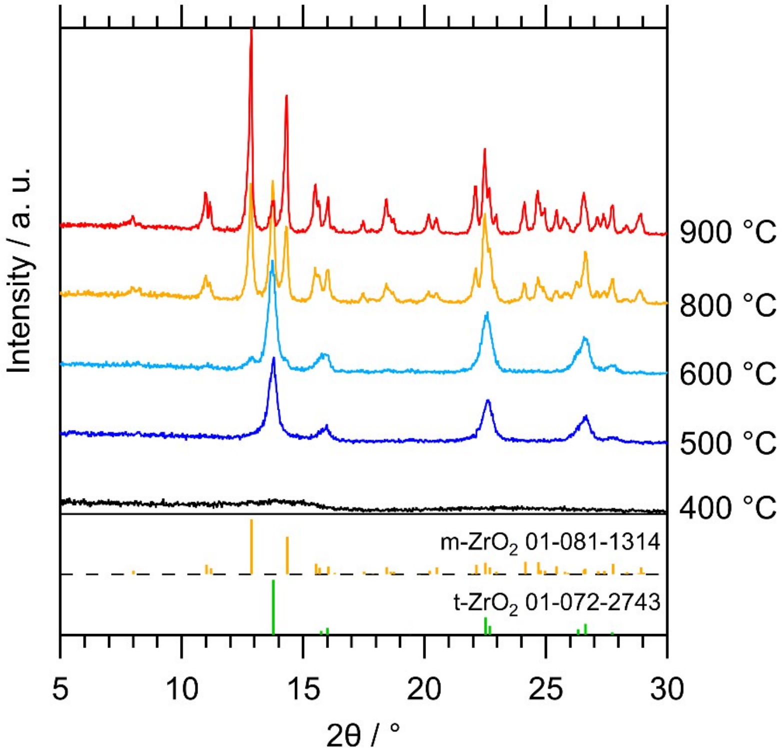

Figure S5: *Ex situ* XRD patterns of the pure ZrO_2 precursor calcined at different temperatures from 400 °C to 900 °C. The polymorph can be tuned from amorphous over tetragonal to monoclinic zirconia (with remnants of t-ZrO_2). The patterns were recorded utilizing Mo $K_{\alpha1}$ radiation (λ = 0.7093 Å). References were taken from the ICDD database.[2,8,9]

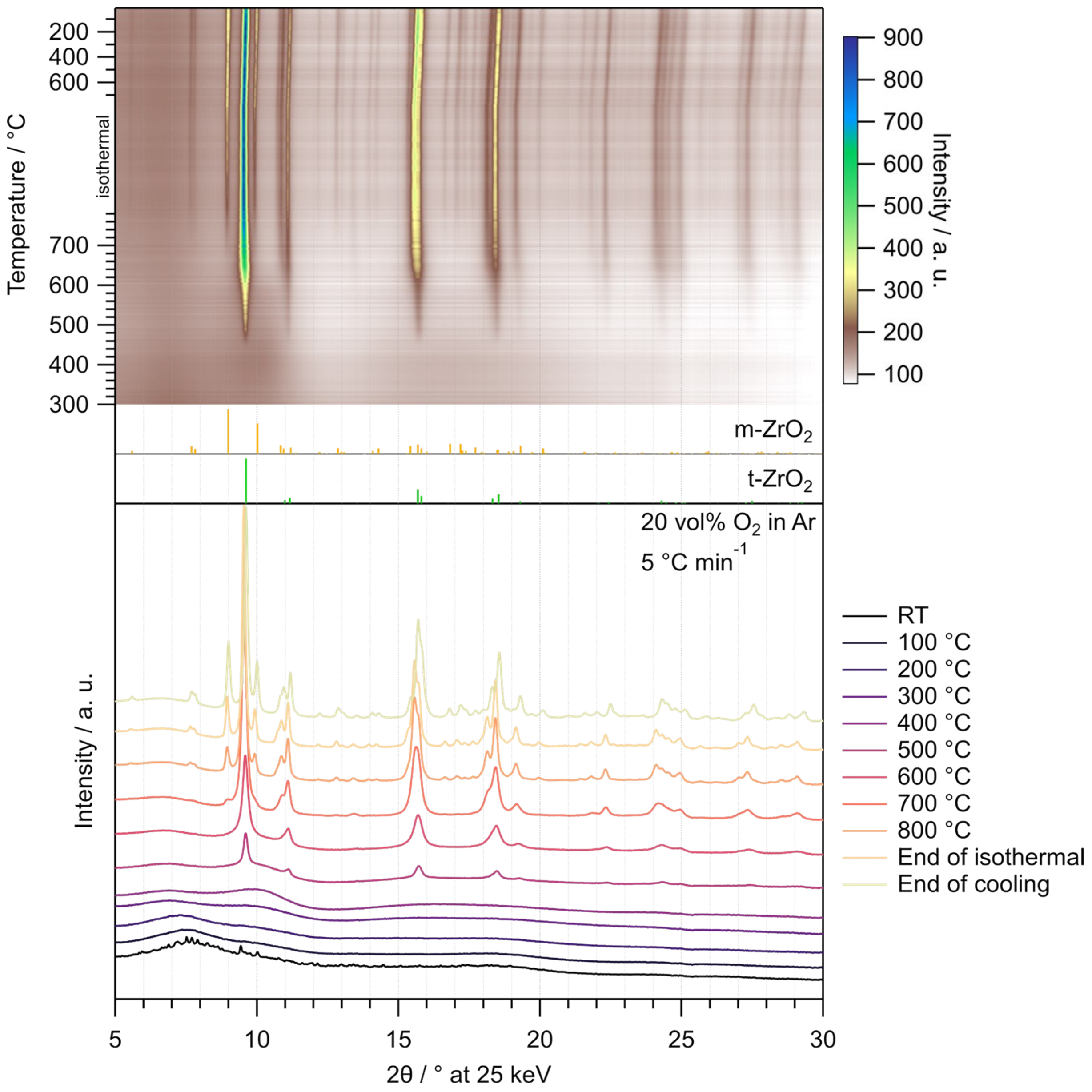

Figure S6: Temperature-resolved *in situ* XRD investigation of the calcination of the pure ZrO_2 precursor after combustion in 20 vol% O_2 in Ar with a heating rate of 5 °C min^{-1} between 25 °C and 800 °C. In the uppermost panel, the entire treatment consisting of heating to 800 °C (depicted from 300 °C), an isothermal period of 30 min and cooling to 25 °C with 20 °C min^{-1} is illustrated as a heat map. Selected diffractograms are depicted in the bottom panel. The patterns were recorded utilizing synchrotron radiation at 25 keV (λ = 0.4959 Å). References were taken from the ICDD database.[2,8,9]

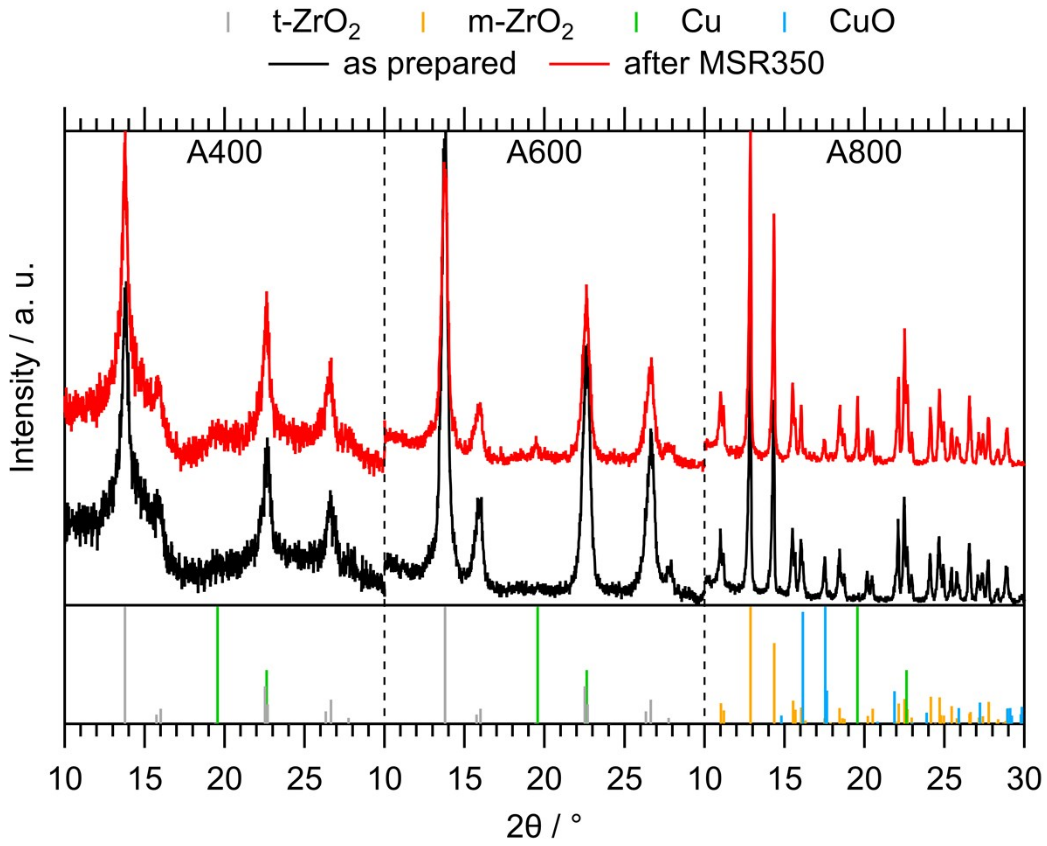

Figure S7: *Ex situ* XRD patterns of the Cu/ZrO_2 samples calcined at different temperatures from 400 °C to 800 °C after calcination (black traces) and after two MSR350 cycles (red traces). The polymorph of zirconia can be tuned from mostly amorphous (A400) to tetragonal (A600) and monoclinic (A800). CuO is only visible in A800 after calcination, whereas A400 and A600 do not exhibit significant reflections of any copper phase. After MSR350, all three systems display the presence of metallic copper. The patterns were recorded utilizing Mo $K_{\alpha 1}$ radiation ($\lambda = 0.7093$ Å). The references were taken from the ICDD database.[2,8–11]

5. MSR on pure ZrO_2 in the recirculating batch reactor including a comparison with the Cu-containing samples

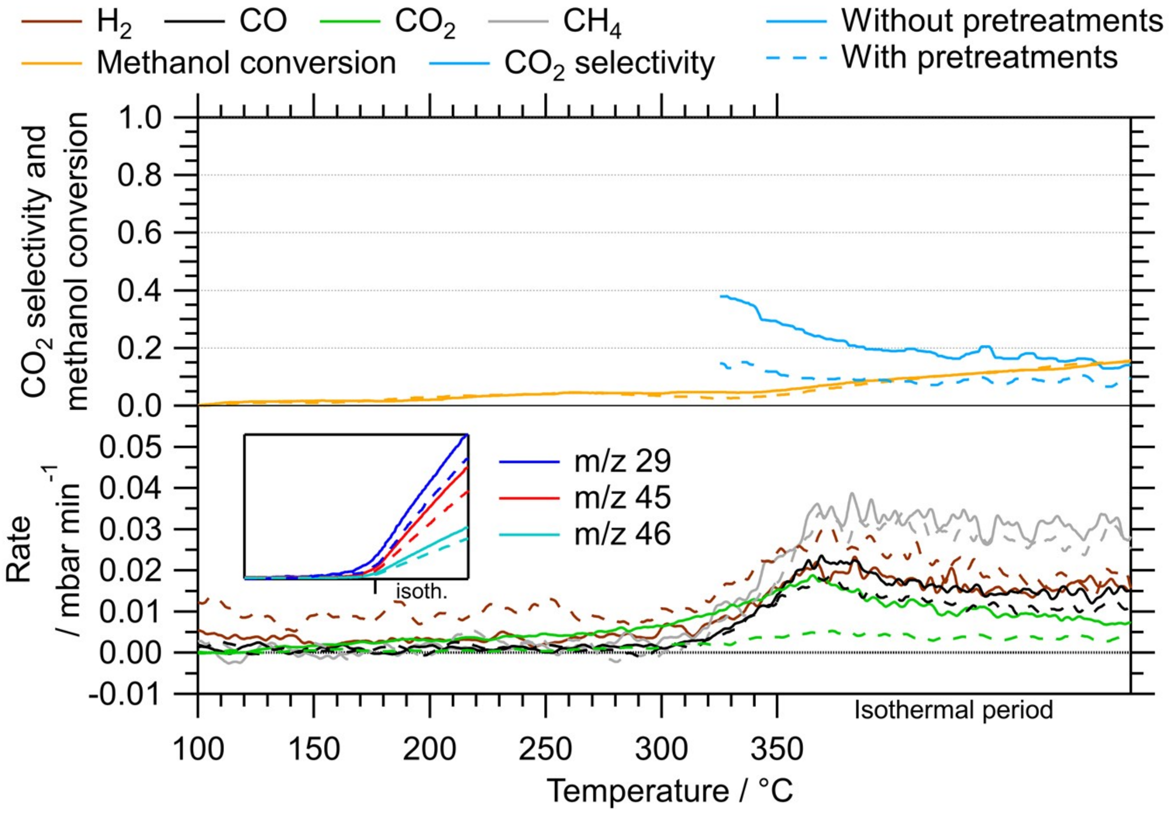

Figure S8: MSR profiles of pure ZrO_2 calcined at 400 °C for 2 h between 100 °C and 350 °C including an isothermal period of 30 min. Colour code: orange – methanol conversion, blue – CO_2 selectivity calculated from the formation rates in mbar min^{-1}, formation rates of brown: H_2, black: CO, green: CO_2, grey: CH_4. The inset depicts the signal (partial pressure) increase of species with mass-to-charge ratios of 29, 45 and 46, which fits the fragmentation pattern of formic acid. The total signal height is comparable to the one of CH_4. Heating rate: 5 °C min^{-1}; sample mass: 19.0 mg.

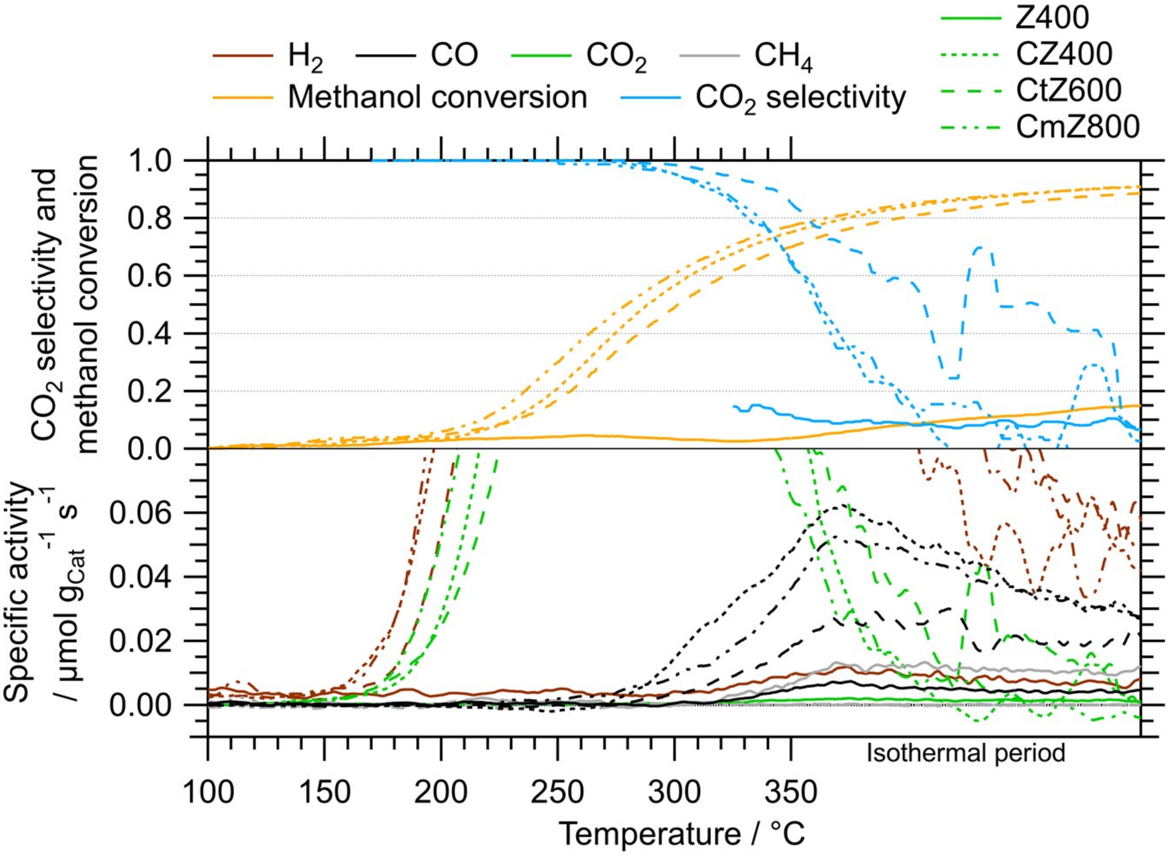

Figure S9: MSR profiles of Z400, CZ400, CtZ600 and CmZ800 between 100 °C and 350 °C including an isothermal period of 30 min. Colour code: orange – methanol conversion, blue – CO_2 selectivity calculated from the specific activities in µmol g_{Cat}^{-1} s^{-1}, specific activity of brown: H_2, black: CO, green: CO_2, grey: CH_4. Heating rate: 5 °C min^{-1}; sample mass: Z400 – 19.0 mg, CZ400 – 18.6 mg, CtZ600 – 21.6 mg, CmZ800 – 20.2 mg.

6. MSR in the continuous flow reactor

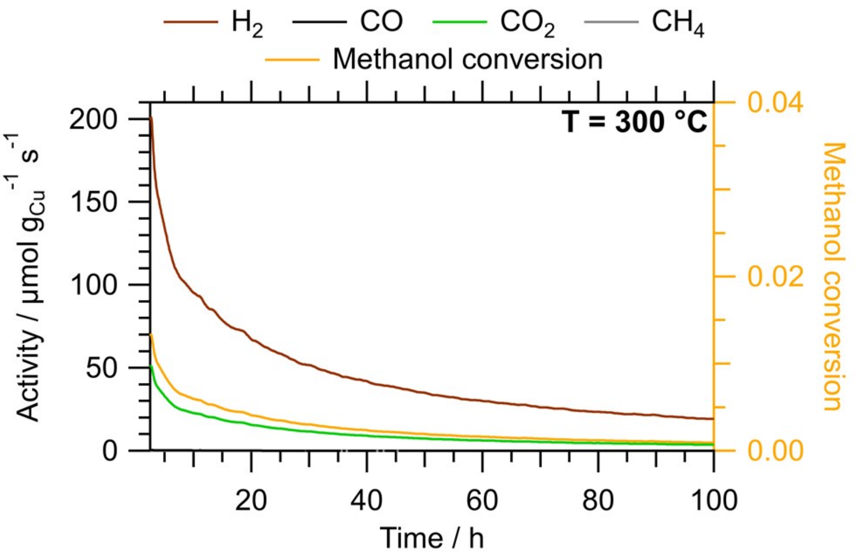

Figure S10: Isothermal long-term MSR test of CtZ600 at 300 °C in a continuous flow reactor with a GHSV of 1750 h^{-1}. Colour code: orange – methanol conversion, specific activity of brown: H_2, black: CO, green: CO_2, grey: CH_4. Due to the deactivation, the activity towards CO formation drops below the detection limit of the GC, rendering the determination of the CO_2 selectivity impossible. Sample mass: 40.1 mg.

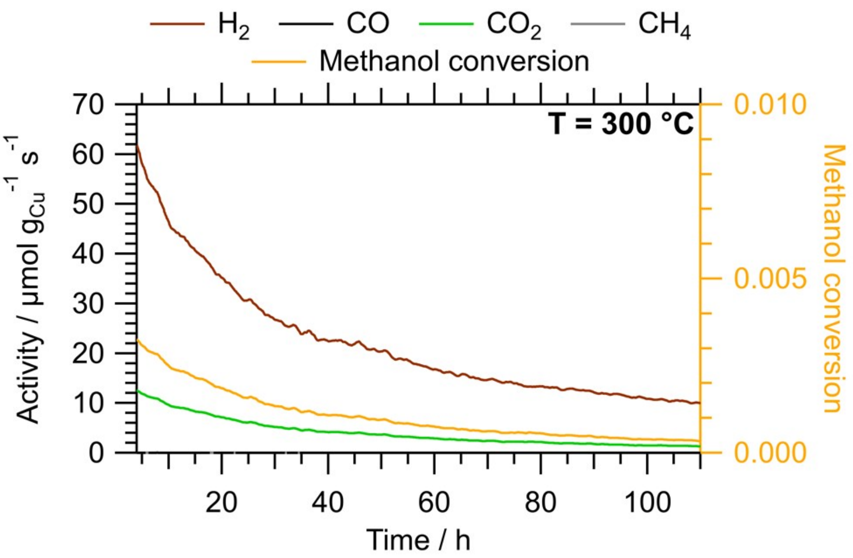

Figure S11: Isothermal long-term MSR test of CmZ800 at 300 °C in a continuous flow reactor with a GHSV of 1750 h^{-1}. Colour code: orange – methanol conversion, specific activity of

brown: H_2, black: CO, green: CO_2, grey: CH_4. Due to the deactivation, the activity towards CO formation drops below the detection limit of the GC, rendering the determination of the CO_2 selectivity impossible. Sample mass: 40.1 mg.

7. Comparison with literature MSR catalysts in the same recirculating batch reactor

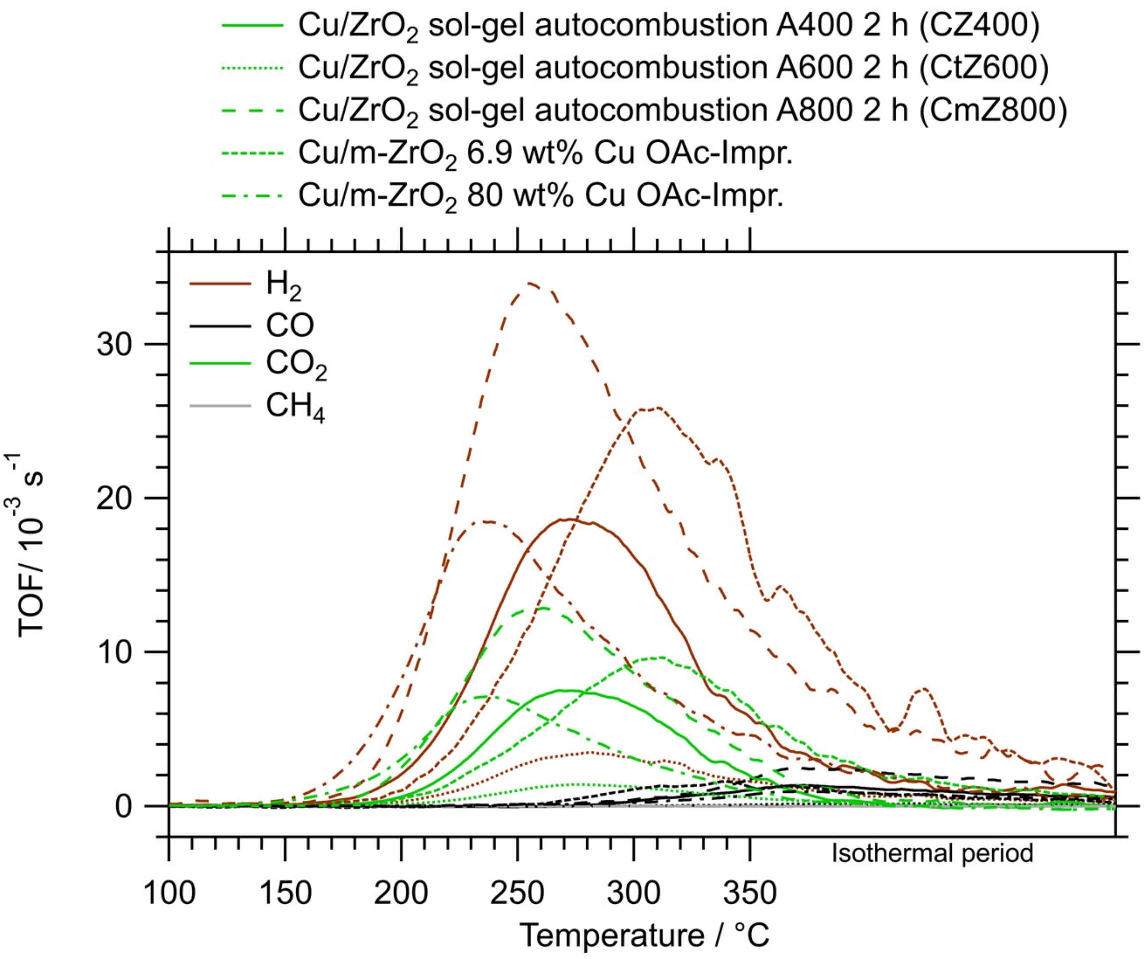

Figure S12: Comparison of CZ400, CtZ600 and CmZ800 with other Cu/ZrO_2 catalysts, one from literature[12] and one analogous system with a higher copper loading, measured in the same recirculating batch reactor setup under identical MSR conditions (100 °C-350 °C; isothermal period of 30 min; heating rate of 5 °C min^{-1}). The turnover frequency was estimated according to the procedure described in section 1.2.

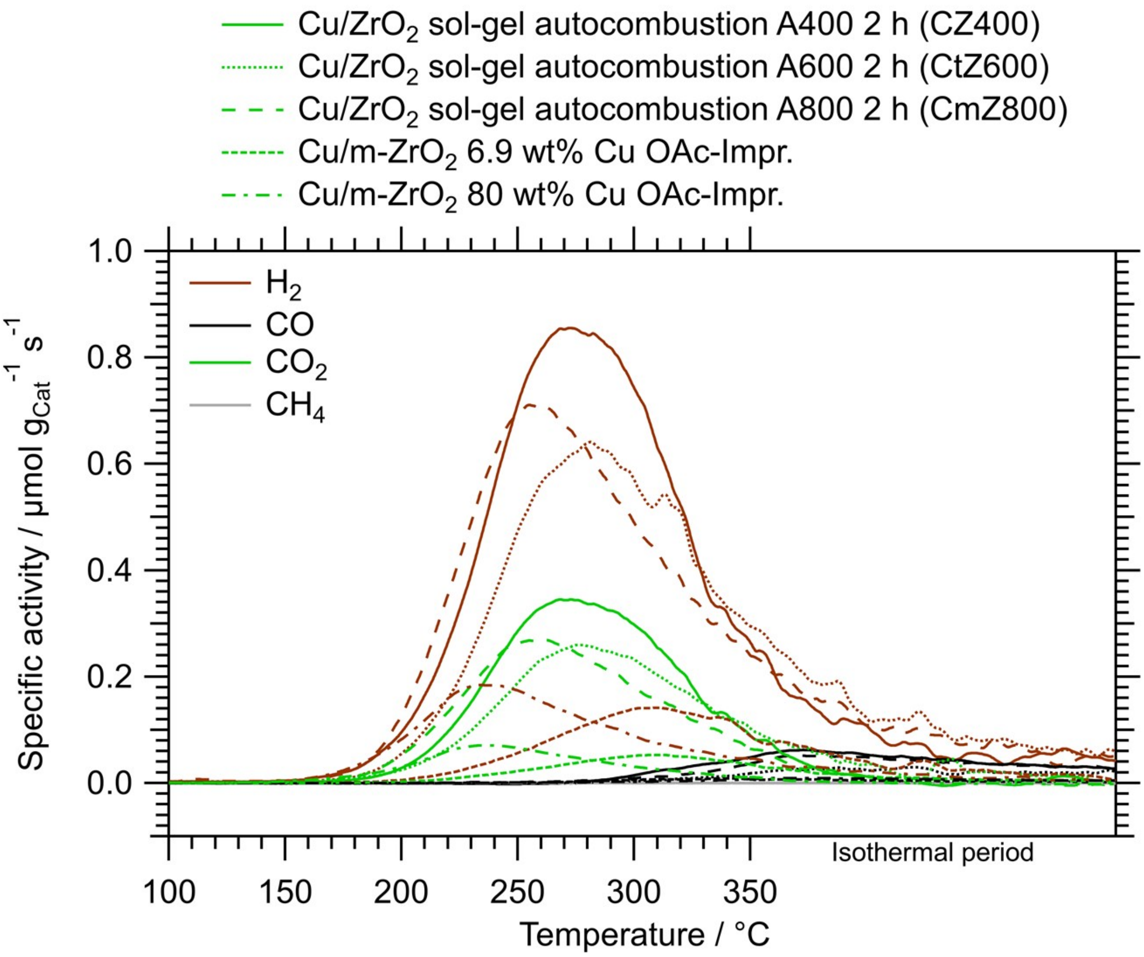

Figure S13: Comparison of CZ400, CtZ600 and CmZ800 with other Cu/ZrO_2 catalysts, one from literature[12] and one analogous system with a higher copper loading, measured in the same recirculating batch reactor setup under identical MSR conditions (100 °C-350 °C; isothermal period of 30 min; heating rate of 5 °C min^{-1}). The rates are expressed in terms of the specific activity per g of catalyst (µmol g_{Cat}^{-1} s^{-1}).

References

1 *WinX*^{POW}, STOE & Cie GmbH, 2008.

2 *ICDD Database PDF-4+*, International Centre for Diffraction Data, Newtown Square, PA 19073, USA, 2010.

3 M. Behrens and M. Armbrüster, in *Catalysis for Alternative Energy Generation*, ed. L. Guczi and A. Erdôhelyi, Springer New York, New York, NY, 2012, pp. 175–235.

4 J. W. Evans, M. S. Wainwright, A. J. Bridgewater and D. J. Young, On the determination of copper surface area by reaction with nitrous oxide, *Appl. Catal.*, 1983, **7**, 75–83. DOI: 10.1016/0166-9834(83)80239-5.

5 *CasaXPS. Processing Software for XPS, AES, SIMS and More*, Casa Software Ltd.

6 W. H. Gries, A Universal Predictive Equation for the Inelastic Mean Free Pathlengths of X-ray Photoelectrons and Auger Electrons, *Surf. Interface Anal.*, 1996, **24**, 38–50. DOI: 10.1002/(SICI)1096-9918(199601)24:1<38::AID-SIA84>3.0.CO;2-H.

7 Y. Nishino, A. R. Krauss, Y. Lin and D. M. Gruen, Initial oxidation of zirconium and Zircaloy-2 with oxygen and water vapor at room temperature, *J. Nucl. Mater.*, 1996, **228**, 346–353. DOI: 10.1016/0022-3115(95)00194-8.

8 C. J. Howard, R. J. Hill and B. E. Reichert, Structures of ZrO_2 polymorphs at room temperature by high-resolution neutron powder diffraction, *Acta Crystallogr., Sec. B: Struct. Sci.*, 1988, **44**, 116–120. DOI: 10.1107/S0108768187010279.

9 H. Boysen, F. Frey and T. Vogt, Neutron powder investigation of the tetragonal to monoclinic phase transformation in undoped zirconia, *Acta Crystallogr., Sec. B: Struct. Sci.*, 1991, **47**, 881–886. DOI: 10.1107/S010876819100856X.

10 H. E. Swanson and E. Tatge, Standard X-ray diffraction patterns, *Natl. Bur. Stand.(US) Circ. 539*, 1953, **1**, 15.

11 S. Asbrink and A. Waskowska, CuO: X-ray single-crystal structure determination at 196 K and room temperature, *J. Phys.: Condens. Matter*, 1991, **3**, 8173–8180. DOI: 10.1088/0953-8984/3/42/012.

12 K. Ploner, M. Watschinger, P. Delir Kheyrollahi Nezhad, T. Götsch, L. Schlicker, E.-M. Köck, A. Gurlo, A. Gili, A. Doran, L. Zhang, N. Köwitsch, M. Armbrüster, S. Vanicek, W. Wallisch, C. Thurner, B. Klötzer and S. Penner, Mechanistic insights into the catalytic methanol steam reforming performance of Cu/ZrO_2 catalysts by *in situ* and *operando* studies, *J. Catal.*, 2020, **391**, 497–512. DOI: 10.1016/j.jcat.2020.09.018.
